# Widespread amyloidogenicity potential of multiple myeloma patient-derived immunoglobulin light chains

**DOI:** 10.1186/s12915-022-01506-w

**Published:** 2023-02-03

**Authors:** Rebecca Sternke-Hoffmann, Thomas Pauly, Rasmus K. Norrild, Jan Hansen, Florian Tucholski, Magnus Haraldson Høie, Paolo Marcatili, Mathieu Dupré, Magalie Duchateau, Martial Rey, Christian Malosse, Sabine Metzger, Amelie Boquoi, Florian Platten, Stefan U. Egelhaaf, Julia Chamot-Rooke, Roland Fenk, Luitgard Nagel-Steger, Rainer Haas, Alexander K. Buell

**Affiliations:** 1 Institut für Physikalische Biologie, Heinrich-Heine-Universität Düsseldorf, Düsseldorf, Germany; 2 Department of Biology and Chemistry, Paul Scherrer Institute, Villigen, Switzerland; 3 Forschungszentrum Jülich GmbH, IBI-7, Jülich, Germany; 4 Department of Biotechnology and Biomedicine, Technical University of Denmark, Lyngby, Denmark; 5 Condensed Matter Physics Laboratory, Heinrich-Heine-Universität Düsseldorf, Düsseldorf, Germany; 6 Department of Health Technology, Technical University of Denmark, Lyngby, Denmark; 7 Mass Spectrometry for Biology Unit, CNRS USR2000, Institut Pasteur, 75015 Paris, France; 8 Cologne Biocenter, Cluster of Excellence on Plant Sciences, Mass Spectrometry Platform, University of Cologne, Cologne, Germany; 9 Department of Hematology, Oncology and Clinical Oncology, Heinrich-Heine Universität Düsseldorf, Düsseldorf, Germany; 10 Forschungszentrum Jülich GmbH, IBI-4, Jülich, Germany

**Keywords:** Amyloid, Immunoglobulin, Multiple myeloma, AL amyloidosis, Light chain, Multiparametric approach

## Abstract

**Background:**

In a range of human disorders such as multiple myeloma (MM), immunoglobulin light chains (IgLCs) can be produced at very high concentrations. This can lead to pathological aggregation and deposition of IgLCs in different tissues, which in turn leads to severe and potentially fatal organ damage. However, IgLCs can also be highly soluble and non-toxic. It is generally thought that the cause for this differential solubility behaviour is solely found within the IgLC amino acid sequences, and a variety of individual sequence-related biophysical properties (e.g. thermal stability, dimerisation) have been proposed in different studies as major determinants of the aggregation in vivo. Here, we investigate biophysical properties underlying IgLC amyloidogenicity.

**Results:**

We introduce a novel and systematic workflow, Thermodynamic and Aggregation Fingerprinting (ThAgg-Fip), for detailed biophysical characterisation, and apply it to nine different MM patient-derived IgLCs. Our set of pathogenic IgLCs spans the entire range of values in those parameters previously proposed to define in vivo amyloidogenicity; however, none actually forms amyloid in patients. Even more surprisingly, we were able to show that all our IgLCs are able to form amyloid fibrils readily in vitro under the influence of proteolytic cleavage by co-purified cathepsins.

**Conclusions:**

We show that (I) in vivo aggregation behaviour is unlikely to be mechanistically linked to any single biophysical or biochemical parameter and (II) amyloidogenic potential is widespread in IgLC sequences and is not confined to those sequences that form amyloid fibrils in patients. Our findings suggest that protein sequence, environmental conditions and presence and action of proteases all determine the ability of light chains to form amyloid fibrils in patients.

**Supplementary Information:**

The online version contains supplementary material available at 10.1186/s12915-022-01506-w.

## Background

Protein aggregates are the hallmark, and in many cases causative agents, of severe disorders, ranging from Alzheimer’s disease to systemic amyloidoses [1, 2]. The loss of protein solubility in these situations that leads to their deposition in various types of aggregates can have multiple origins, such as point mutations, post-translational modifications, and over-production of proteins. The latter phenomenon occurs for example in multiple myeloma (MM), a malignancy characterised by a clonal expansion of abnormal plasma cells in the bone marrow [3]. In the healthy state, they produce a complete immunoglobulin and in some cases additionally the corresponding monoclonal light chain (LC) [4]. Approximately 15% of the patients with MM produce exclusively light chains [5]. The 25-kDa-sized light chain proteins are usually either excreted or degraded by the kidney, but high monoclonal quantities and low renal clearance can lead to the deposition at various sites within the kidney [6]. The deposits contain diverse kinds of IgLC aggregates, which cause different diseases, such as light chain deposition disease (LCDD), where the aggregates have an amorphous nature [7] and AL amyloidosis, where aggregates consist of amyloid fibrils [8].

Currently, there is no possibility to accurately predict the in vivo solubility and deposition behaviour of a particular monoclonal light chain found in the blood or urine of a patient. A large number of studies has been performed in recent years in order to understand the in vivo solubility and aggregation behaviour of a patient-derived light chain through a detailed in vitro investigation of the biophysical and biochemical properties [9–13]. In particular, the question as to which properties distinguish the amyloid fibril forming light chains from those that do not form amyloid fibrils in vivo has been extensively studied.

Various molecular properties of the light chains, such as thermal stability [9, 14–16, 16–19] tendency to dimerize [20–22] and the dimer interface [23], or protein dynamics [14, 24], have been proposed to correlate with their deposition as amyloid fibrils. However, some findings are contradictory, for instance the reports that dimeric LCs are more often found in AL patients [20, 22] versus those that the monomeric state is more critical for fibril formation [21] and that stable dimers can even inhibit the aggregation [25]. Hence, no clear picture has as yet emerged as to which factors define IgLC amyloidogenicity in vivo.

Each patient-derived light chain protein has a unique amino acid sequence determined by somatic (V(D)J) recombination and various somatic mutations [26]. Somatic hypermutations affect mainly the complementarity-determining regions (CDRs) in the variable domain with a rate of approx. 10^−3^ mutations per base pair per cell division [27]. Light chains occur either as a λ or a κ isotype, which are encoded by the immunoglobulin λ (IGL) [28] or the κ (IGK) [29] locus. The majority of light chain amyloidosis cases are associated with λ light chains [30] with an over-representation of five different germline genes (IGLV1-44, IGLV2-14, IGLV3-1, IGLV6-57 and IGKV1-33) [31, 32]. This finding suggests some connection between sequence and the amyloid propensity of an IgLC.

The sequence of an IgLC can either be obtained through DNA sequencing of tissue from bone marrow biopsies [33], or through de novo protein sequencing by mass spectrometry [34]. The latter, however, is challenging because of the absence of the sequence under study in the databases usually employed in mass spectrometry (MS)-based proteomics [35].

In order to investigate the link between light chain sequence and aggregation behaviour, we have developed a complete de novo protein sequencing workflow based on a combination of top-down [36] and bottom-up proteomics with specific data analysis for patient-derived light chains [37]. The light chains investigated in this study were derived from 10 patients presenting light chains in their urine (2 isotype lambda and 8 isotype kappa) and represent a sub-set of a larger collection from a patient cohort at the University Hospital Düsseldorf, which has already been subjected to an initial biochemical and biophysical characterisation [13]. The inclusion criterion of a given sample into the present study was the availability of the amino acid sequence [37]. The detailed Thermodynamic and Aggregation Fingerprinting (ThAgg-Fip) was carried out for those samples (9/10) that had sufficient concentration and purity to allow for well-defined biophysical and biochemical experiments. ThAgg-Fip consists of the characterisation of various IgLC features that have been shown to correlate with amyloid fibril formation in patients, e.g. dimerisation, thermodynamic stability (thermal and chemical), thermally and chemically induced aggregation (amorphous and amyloid) and their respective concentration dependencies, and enzymatic digestability. ThAgg-Fip represents probably the most comprehensive and detailed characterisation of disease-related IgLCs to date.

The 9 samples for which ThAgg-Fip was performed are obtained from MM patients without evidence of amyloid fibril formation. Consequently, our study was mostly carried out on light chain proteins, which would generally be considered to be non-amyloidogenic. However, we find that all proteins of our study are observed to form amyloid fibrils in vitro under conditions of acidic pH, where the present (co-purified) proteases (mostly cathepsins) led to the rapid digestion of the light chains into shorter fragments. We were able to determine the sequence regions involved in the formation of fibrils for some of our samples and found no correspondence with aggregation hot spots predicted by commonly used algorithms.

Our findings challenge the current paradigm that the origin of the amyloidogenicity is to be found in the amino acid sequence of the light chains alone. We conclude that many IgLCs, in particular also many of those that are not observed to form amyloid in patients, have intrinsic amyloidogenicity. The interplay between protein sequence, environmental conditions and presence and action of proteases defines whether a given light chain deposits in the form of amyloid fibrils in a given patient, and therefore the phenomenon of IgLC aggregation defies mono-causal explanations.

## Results

**Fig. 1:**
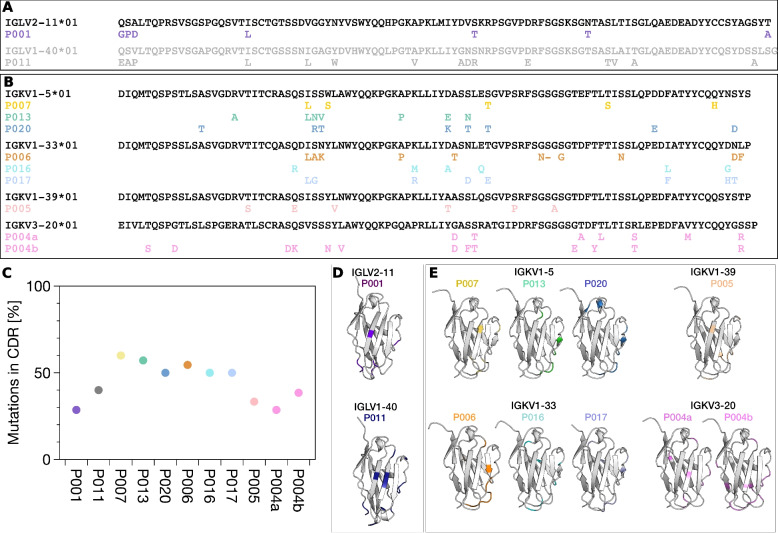
Sequences of the **A** λ and **B** κ variable domain of the germline sequences. The mutations in the sequences of the IgLC samples compared to the corresponding germline sequence are labelled. The relative proportion of mutations located in the CDRs are plotted (**C**). Three-dimensional structural models of the variable regions of the λ (**D**) and κ (**E**) are visualised with PyMOL 2.4 (Schrödinger). The mutated amino acids in the investigated IgLCs are highlighted in colour

This study is based on the detailed biochemical and biophysical characterisation of patient-derived IgLCs which were purified from urine samples of patients with a monoclonal light chain disease. These IgLCs were selected from the samples of a previous study [13]. We have recently developed a novel highly efficient mass spectrometry workflow, combining both top-down and bottom-up proteomics, that allows us to determine both the amino acid sequences of these IgLCs, as well as the dimerisation behaviour [37]. Figure 1 shows the sequences and structures of the variable domains of these IgLCs to give an illustration of their sequence differences compared to the germline, and where in the structure the amino acid changes are situated. The structures were obtained through modelling, starting from the published structures of the corresponding germline sequences (see ‘Methods’ section for details). The alignment of the full-length sequences with the marked framework regions (FR) and complementarity-determining regions (CDRs) can be found in the supplement (Additional file 1: Fig. S1 and Additional file 1: Fig. S2). P011 is the only IgLC sample that stems from a confirmed amyloidosis patient. However, P011 contained a high concentration of contaminating proteins, which were not IgLCs but had a similar molecular weight as the IgLC, such that ThAgg-fingerprinting was not performed for this IgLC.

We set out to perform a comprehensive characterisation of the thermodynamic and aggregation properties of these patient-derived IgLCs. To this end, we have combined a wide array of state of the art biophysical and biochemical methods into the ThAgg-Fip approach. We included the experimental parameters that have previously been reported to correlate with light chain amyloidogenicity, such as dimerisation, thermal stability and proteolytic digestibility. Furthermore, we included a detailed characterisation of the thermodynamics and aggregation behaviour of each light chain. The resulting ThAgg fingerprints are listed in Table 1.

**Table 1:** Overview over the thermodynamic and aggregation fingerprints of the IgLCs of this study. All *T*_m_ values, turbidity maxima and aggregate sizes are reported at a concentration of ca. 30 μM. The DSF experiments at pH 5 (pH 7.4) were performed at a scan rate of 2.5°C/min (1.0°C/min). Data on *T*_m_ and refolding fraction by DSC and trypsin digestibility are from [13]. The concentration dependence of the melting and aggregation temperatures was explored in the range between ca. 15 and 120 μM. *m-value fixed for all samples. **Different m-value allowed in fit. ***Only one population with intermediate *s*-value detected

	P001	P004	P005	P006	P007	P013	P016	P017	P020
Dimerisation by SDS-PAGE	0.96	0.71	0.43	0.3	0.32	0.36	0.49	0.4	0.52
Dimerisation by MS	1	0.97	0.35	0.07			0.9	0.3	0.56
Dimerisation by AUC	1	0.94	0.55	0.1	***	***	0.72	0.15	0.91
*T*_m_ DSF (pH 5) [°C]	63.7	51.1	51.0	54.9	50.3	51.7	54.1	50.4	56.3
*T* (max. turb. @ pH 5) [°C]	> 90	62	71	No turb.	66	64	No turb.	65	83
*T*_m_ DSC (pH 7.4) [°C]	64.6	58.5	51.2	50.4	55.4	52.2	56.8	51.7	53.5
*T*_m_ DSF (pH 7.4) [°C]	62.6	54.2	50.9	50.6	53.8	52.6	54.7	49.4	50.4
*d T*_m_/*d* log(c) (pH 7.4)	−0.33	−1.19	−1.20	0.9	−0.55	−1.10	−0.17	0.15	0.23
*T*_agg_ DSF (pH 7.4) [°C]	54.9	53.0	50.8	59.4	55.2	50.2	n/a	61.7	50.3
*d T*_agg_/*d* log(c) (pH7.4)	−5.8	−2.9	−4.1	−1.5	−3.2	−4.1	−0.5	−22.0	−4.6
Cumul. rad. (pH 7.4, 70°C) [nm]	>1000	>1000	~1000	7	~300	~25	5.5	16	>1000
Fract. refold - DSF (no urea)	Small	Small	Small	Large	Small	Small	Medium	Large	Small
Fract. refold - DSF (2 M urea)	0	> 50	> 50	> 50	> 50	< 50	< 50	> 50	< 50
Fract. refold - DSC	0	0.18	0.36	0.68	0.33	0.38	0.41	0.7	0.22
ΔG [kJ/mol] at 37°C	26.3±1.5	25.3±0.4	20.8±2.1	20.8±0.8	23.7±0.6	21.2±0.7	10.0±0.7	21.8±1.1	20.2±0.5
m-value	7.1*	7.1*	7.1*	7.1*	7.1*	7.1*	2.3±0.5**	7.1*	7.1*
Digestibility by trypsin	0.1	1	0.7	0.24	1	1	0.17	0.98	1
Acid induced amyloid form	Yes	Yes	Yes	Yes	Yes	Yes	Yes	Yes	Yes

### Dimerisation of the light chains

We first set out to robustly characterise the monomer-dimer distribution of the IgLCs. The tendency of disease-related over-produced immunoglobulin light chains to dimerise has previously been proposed to correlate with the protein’s tendency to form amyloid fibrils [20]. In a previous study, we determined the degree of dimerisation with non-reducing SDS-PAGE gels [13]. Here we aimed to improve our previous results by both sedimentation velocity analytical ultracentrifugation (AUC), as well as mass spectrometry. AUC allows the analysis under native conditions in solution and mass spectrometry allows reliable identification of protein monomers and multimers. A detailed discussion of the dimerisation behaviour derived from MS analysis, including the observation of mixed dimers formed from two IgLC isoforms, can be found in [37]

**Fig. 2:**
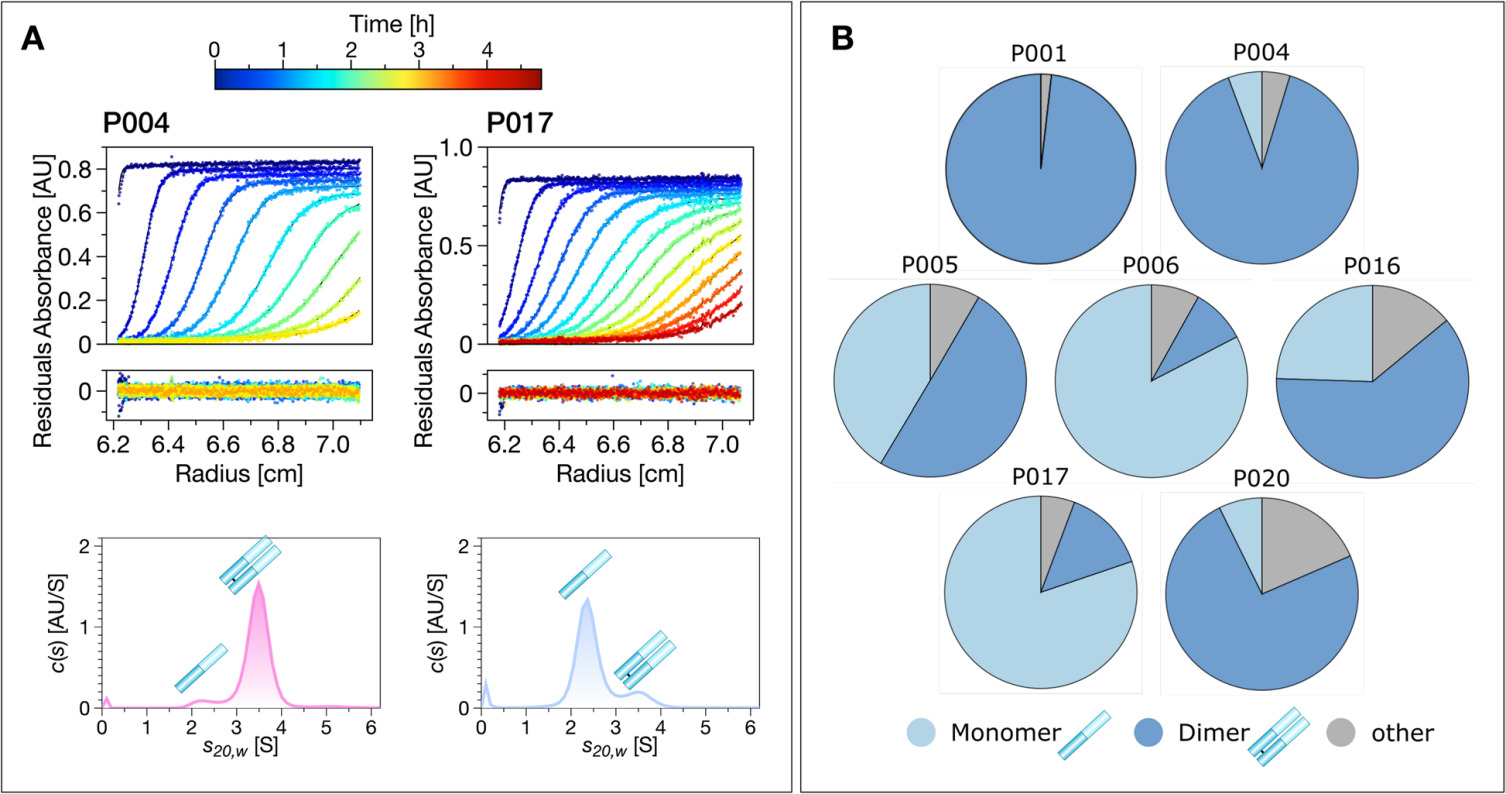
IgLC dimerisation probed by sedimentation velocity analytical ultracentrifugation (SV-AUC). **A** Examples of raw data from SV-AUC runs of P004 and P017. The distribution of sedimentation coefficients *c*(*s*) is shown at the bottom. **B** The monomer (light blue), dimer (dark blue) and other (grey) content measured by AUC

The fractions of monomer and dimer displayed in Fig. 2 are extracted from sedimentation velocity *c*(*s*) profiles. Both species could be fitted as two distinct distributions in the displayed samples. As ‘other’, we denote additional contents of some of the samples, for example HSA (in P020) or aggregates.

The measured average *s*_*20*,w_-value for the monomer is 2.32±0.07 S and for the dimer 3.53±0.18 S. The *c*(*s*) profiles of P007 and P013 displayed only one species situated between the s-value of a monomer and a dimer. We also find that the dimers can differ surprisingly strongly in their form factors, ranging from close to globular to strongly elongated (see Additional file 1: Fig. S3).

Taken together, these techniques provide a very accurate picture of the monomer-dimer distribution of the IgLCs in solution. None of the examined IgLCs occurs exclusively in its monomeric form, but P001 is exclusively dimeric. Overall, our samples span a wide range of degree of dimerisation (~0.1–1). The results of the three different methods, which are in good agreement, are listed in Table 1. It should be noted here that our method of purification by size exclusion chromatography (SEC; see ‘Methods’ section and Additional file 1: Fig. S4) has the potential to introduce a degree of bias into the observed monomer-dimer distribution, but is unlikely to change the conclusion that our IgLC samples span virtually the full range of observable dimerisation behaviour.

### Thermal and chemical stability and thermally induced aggregation

**Fig. 3:**
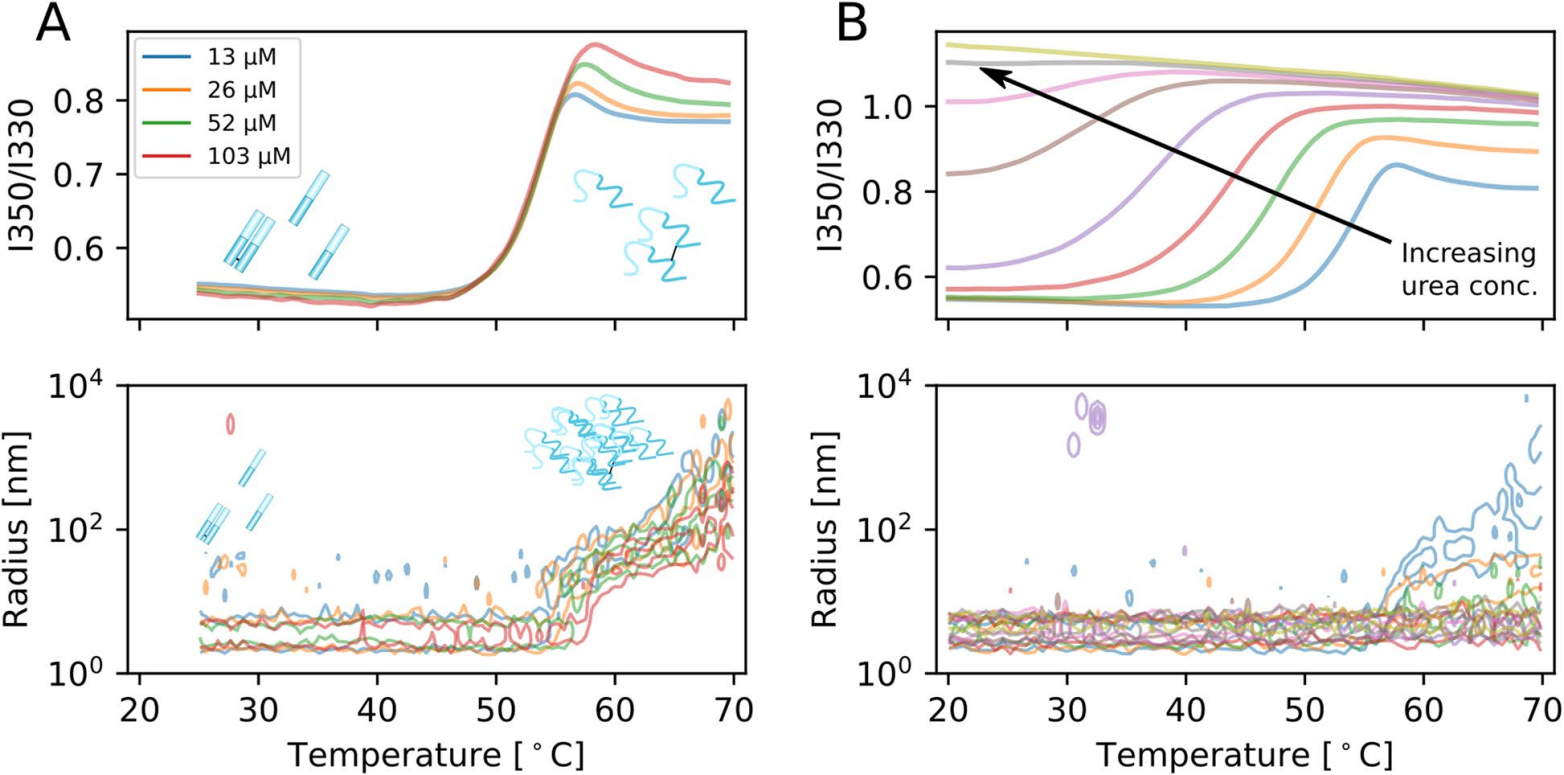
Thermal and chemical stability of IgLCs. **A** Thermal unfolding of P007 (as an example) at 103, 52, 26 and 13 μM followed by intrinsic fluorescence. In addition, the evolution of the distribution of hydrodynamic radii is shown as a function of temperature as a contour plot (full data set with all samples in Additional file 2: Fig. S1. Contours are spaced with 0.1 and the lowest contour drawn is at 0.03 amplitude. **B** Combined chemical and thermal unfolding of P007 (as an example) at 20 μM and urea concentrations of 0 to 5.36 M in 8 steps (full data set with all samples in Additional file 2: Fig. S2)

We then proceeded to characterise the thermal and chemical stability as well as aggregation behaviour of the IgLC samples at neutral pH (7.4). We performed thermal ramping experiments (25–70°C) at different protein concentrations (see ‘Methods’, Additional file 2: Fig. S1) while simultaneously measuring the intrinsic Trp fluorescence of the IgLC samples as well as performing dynamic light scattering (DLS) experiments. An example of such experiments (for P007) is shown in Fig. 3a. At the beginning of the experiment, the native protein (monomeric and dimeric) dominates the DLS signal in all cases.

We found a dependence of both the unfolding temperature (*T*_m_), as well as the temperature of onset of aggregation (*T*_agg_), on the protein concentration, and we quantify these dependencies by defining the parameters *d T*_m_/*d* log(c) and d *T*_agg_/*d* log(c). While *d T*_agg_/*d* log(c) is always negative, we found that *d T*_m_/*d* log(c) can be both negative and positive. It has been reported that aggregation induced by unfolding can lead to a concentration-dependent decrease in unfolding temperature according to the law of mass action [38], whereas molecular crowding can have the opposite effect, i.e. a concentration-dependent stabilisation [39]. The onset and degree of aggregation vary considerably between the samples. Aggregation can start significantly before (e.g. P001) or after (e.g. P006) the midpoint of unfolding. Furthermore, the aggregate sizes (as quantified from the cumulant radius of the samples at 70°C) vary between sizes below 10 nm (e.g. P016) and sizes of more than 1 μm (e.g. P020). We also followed the degree of refolding by cooling the samples down and found that most samples (except P006 and P017) show very little refolding under these conditions, in agreement with previously published data from differential scanning calorimetry (DSC) experiments (Table 1).

Next, we extended these measurements by performing a temperature ramp of samples at different denaturant (urea) concentrations (see Additional file 2: Fig. S2); examples are shown in Fig. 3b, where the fluorescence ratio (350 nm/330 nm) and the DLS size distributions are shown. As expected, the proteins become increasingly destabilised at higher temperatures and urea concentrations. Interestingly, we find that significant aggregation is only observed in the absence of urea, as well as for the 1–2 lowest urea concentrations. This information allowed us to perform a global fit to a model of the temperature-dependent IgLC thermodynamic stability [40], where we excluded the samples where aggregation was observed. Details of the modelling can be found in the ‘Methods’ section. All the global fits can be found in Additional file 2: Fig. S3 and Additional file 2: Fig. S4. While this analysis allows to define the stability at any temperature in the measured interval, we report only the apparent folding free energy, ΔG, at 37°C as a measure for the protein stability under physiological conditions in Table 1, together with the other data on thermal stability and aggregation discussed above. Despite the lack of visible aggregation under the urea concentrations used for our fits, the unfolding was only partly reversible upon cooling down, albeit significantly better than in the absence of urea. We have divided the degree of refolding (at 2 M urea) into three categories: more than 50% refolding (5/9 IgLCs), less than 50% refolding (3/9 IgLCs) and no significant refolding (1/9 IgLCs), and this information is tabulated in Table 1. Urea therefore both prevents aggregation and promotes correct refolding of the monomeric/dimeric soluble state. The higher the degree of refolding, the more closely the apparent folding free energy, ΔG, corresponds to the true thermodynamic folding free energy. Values determined for low degrees of refolding need to be therefore interpreted with care, but we nevertheless quote the results of our global analysis in the table for all IgLCs.

One of the most intriguing and defining features of IgLCs is their temperature-induced aggregation behaviour at mildly acidic pH [5], which has historically been used for the diagnosis of free IgLCs in urine (Bence-Jones proteins) in multiple myeloma and related diseases [41]. Upon heating to ca. 60°C, most free IgLC-containing urine samples become turbid, and upon further heating to above 90°C, the turbidity is partly reversible in many cases (Additional file 2: Fig. S5). This method, the physical basis of which is not very well understood, was eventually replaced by more modern methods, because of the limited reliability. Based on the very different aggregation behaviour of our IgLCs at neutral pH, we decided to simulate the original diagnostic method by performing rapid thermal ramping experiments from 25 to 90°C in microcapillaries at a standardised protein concentration (see ‘Methods’ section for details) and found indeed that the majority of the samples showed a decrease of turbidity above ca. 70°C (Additional file 2: Fig. S5). However, the turbidity, its temperature maximum and the degree of its reversibility varied greatly between the samples. The simplicity and practical relevance of this experiment, the diversity of observed behaviour and the current lack of a mechanistic explanation of the heat-induced reversal of aggregation prompted us to include this data into our comprehensive biophysical characterisation.

### LC aggregation at acidic pH values

**Fig. 4:**
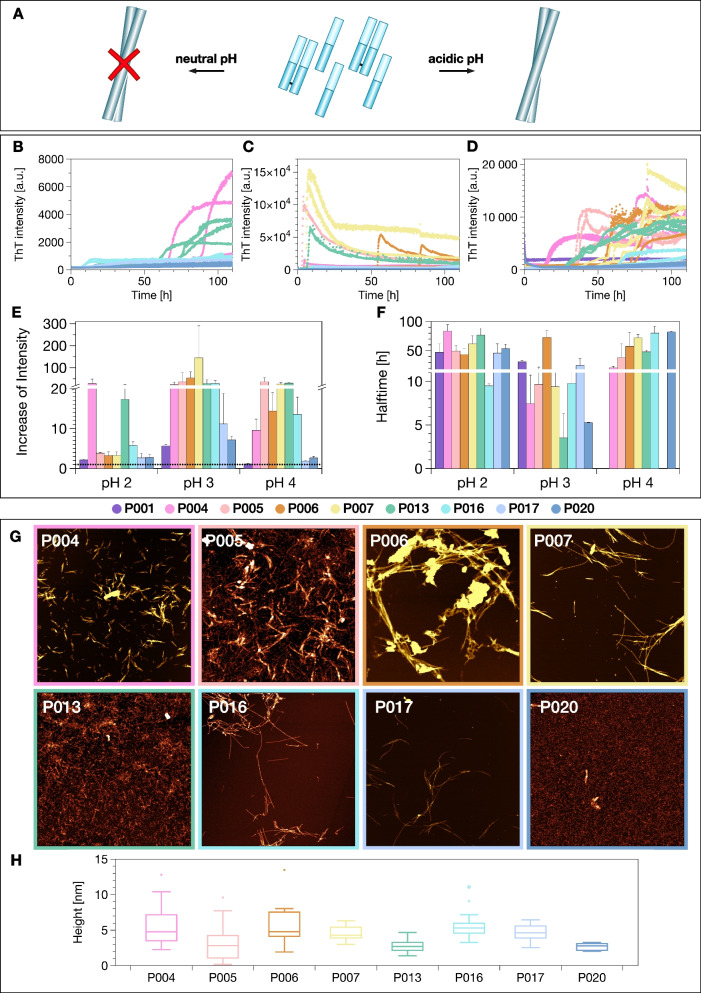
Incubation of the IgLCs at acidic pH leads to the formation of amyloid fibrils. **A** Incubation of the IgLCs of this study at neutral pH does not lead to the formation of amyloid fibrils [13], whereas incubation at acidic pH (≤4) does. ThT fluorescence aggregation assay of the IgLC samples at **B** pH 2, **C** pH 3 and **D** pH 4 monitored in high-binding plates in the presence of glass beads under conditions of mechanical agitation. The aggregation kinetics are analysed by the **E** increase of intensity and **F** aggregation halftime. **G** AFM height images of IgLC samples after aggregation at pH 2. The image size is 5 × 5 μm. The colour range represents the height from −2 to 15 nm. **H** Box plot of the observed values of the height is illustrated.

In our previous study, we found that some of the investigated light chains formed amyloid fibrils at acidic pH values (pH 4), even though they came from patients without confirmed AL amyloidosis [13]. As we had furthermore detected a strong influence of the nature of the reaction vessel surface on the aggregation kinetics, we decided to examine the aggregation behaviour of the IgLC in the present study more systematically, at pH 2, pH 3 and pH 4 in high-binding polystyrene multi-well plates (Fig. 4). Almost all samples and conditions displayed an increase in ThT fluorescence intensity, but the relative increase in the fluorescence intensity was found to differ significantly between the samples. The most efficient aggregation was detected at pH 3.

In Fig. 4G, AFM height images of products from the aggregation experiment at pH 2 are shown.

Additional AFM images of fibrils formed in different reaction vessels can be found in Additional file 3: Fig. S1.

**Fig. 5:**
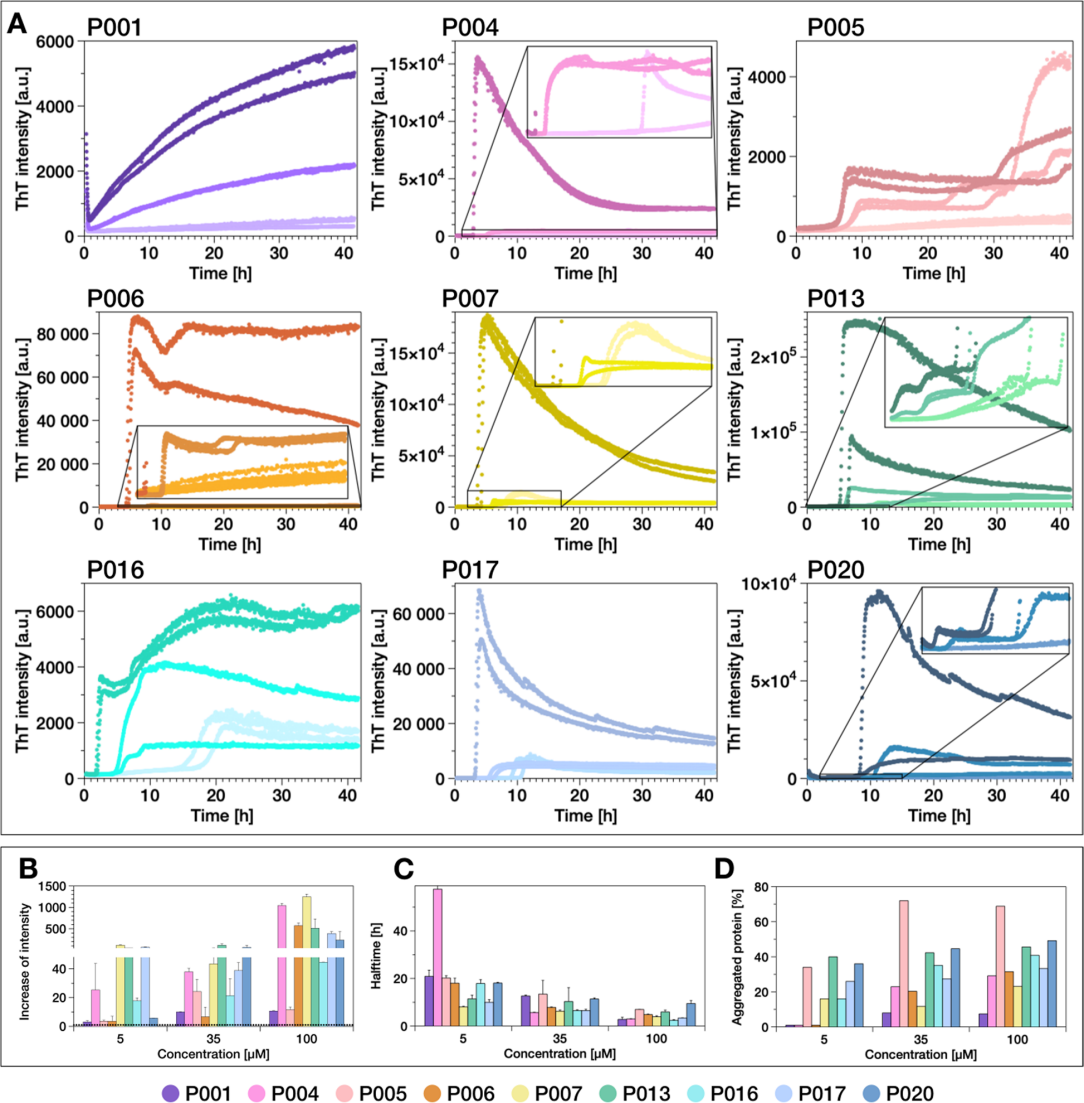
**A** ThT fluorescence aggregation assay of the IgLC samples at pH 3 in the absence of glass beads measured in high-binding surface plates under agitation conditions. The different colour shades indicate the different monomer concentrations: light 5 μM; intermediate 35 μM; dark 100 μM. Overview of the effect of pH 3 on IgLC aggregation assayed by **B** increase of ThT fluorescence intensity, **C** the halftime of the aggregation and **D** relative fraction of aggregated protein determined by measuring the remaining soluble content after centrifuging the end product of the aggregation reactions. The three replicates per condition were combined before centrifugation

Since the aggregation at pH~3 is the most rapid, we examined the aggregation behaviour of the IgLCs at different monomer concentrations (5, 35 and 100 μM) under this pH condition (Fig. 5). The plate was shaken in the absence of glass beads. Apart from P001, whose aggregation had no lag time, the aggregation kinetics followed the typical sigmoidal time course of amyloid formation.

After reaching the steady-state phase, the ThT-signal was found to decrease in the case of the samples at 100 μM concentrations. This decrease can be explained by the formation of insoluble, big aggregates, which sediment and disappear out of the focus of detection.

We tested the influence of glass beads and pre-formed fibrils (seeds) on the aggregation kinetics of P016 in more detail as an example (Additional file 2: Fig. S2). At this condition, even though the seeds accelerated the fibril formation significantly, a lag time was still observed. If the proteins were pre-incubated at acidic pH, on the other hand, rapid aggregation was observed from the moment of addition of the seeds at pH 3.

The influence of the pre-incubation of the monomer at acidic pH suggests a possible modification that the soluble IgLCs might undergo under these conditions. We therefore set out to probe whether the IgLCs can undergo proteolysis. For this purpose, the samples were incubated in an Eppendorf tube at 37°C under quiescent conditions, in order to slow down as much as possible the formation of amyloid fibrils. The samples were analysed at different time points using SDS-PAGE and the microfluidic diffusional sizing [42] device Fluidity One (Additional file 3: Fig. S3). The time course of the relative proportions of native IgLCs (monomer and dimer combined) was determined by SDS-PAGE. Incubation at pH 3 was found to have a strong effect on the size of the IgLCs; already after 1 h incubation, almost no intact protein was found to remain. Only P001, which occurs exclusively in dimeric form, partly resisted acid induced degradation over several hours. At pH 4, the samples were found to be more resistant, but after an incubation of 24 h the IgLCs were found to be fragmented to between 50 and 100%. The fragmentation at pH 2 was found to be faster than that at pH 4, but slower than at pH 3.

The observed decrease in concentration can be explained by the fact that amorphous aggregates as well as fibrils are not detected in the same quantitative manner by the Fluidity One instrument, because large aggregates may not be able to enter the microfluidic channels. Furthermore, aggregates may not be as efficiently stained by the fluorescent modification used for protein quantification in the Fluidity One (Additional file 3: Fig. S3C).

Such an efficient fragmentation of the IgLCs cannot easily be explained by acid-catalysed hydrolysis of the polypeptide backbone. We therefore searched for the possible presence of proteases using a regular MS-based proteomics approach. The results indeed revealed the presence of different cathepsins in the IgLC samples purified from patients’ urine (see Table 2). The activity of cysteine cathepsins is increased at acidic pH values and they are mostly unstable and inactive at neutral pH [43, 44].

**Table 2:**
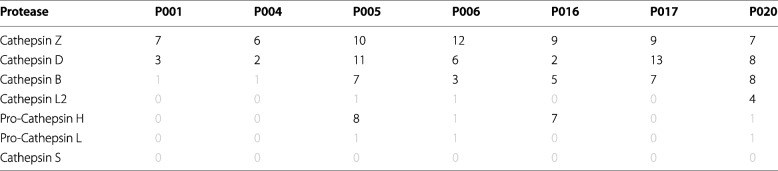
List of different cathepsins which were found in the samples. The MS/MS count represents the number of MS/MS spectra leading to an identified protein. The black numbers indicate a confident identification of the protein. Proteins, which are only identified through one peptide, are not validated (number coloured in grey)

In order to probe whether the fragmentation of the IgLC at acidic pH values was caused by the present cathepsins, we incubated the samples with protease inhibitors. As inhibitors, we choose E-64 and pepstatin A. E-64 (trans-Epoxysuccinyl-L-leucylamido(4-guanidino)butane) is an active-site directed, irreversible inhibitor of cysteine proteases and is known to inhibit cathepsin B, cathepsin L, cathepsin H, and cathepsin Z [45]. Pepstatin A (isocaleryl-L-valyl-L-valyl-4-amino-3-hydroxy-6-methylheptanoyl-L-alanyl-4-amino-3-hydroxy-6-methylheptanoic-acid) is a very selective and potent inhibitor of cathepsin D, which is one of the major aspartyl endopeptidases in mammalian cells and has a pH optimum at 3.5–5 [46]. Cathepsin D was the second most frequent cathepsin present in the samples (in P017 it had the highest fraction).

**Fig. 6:**
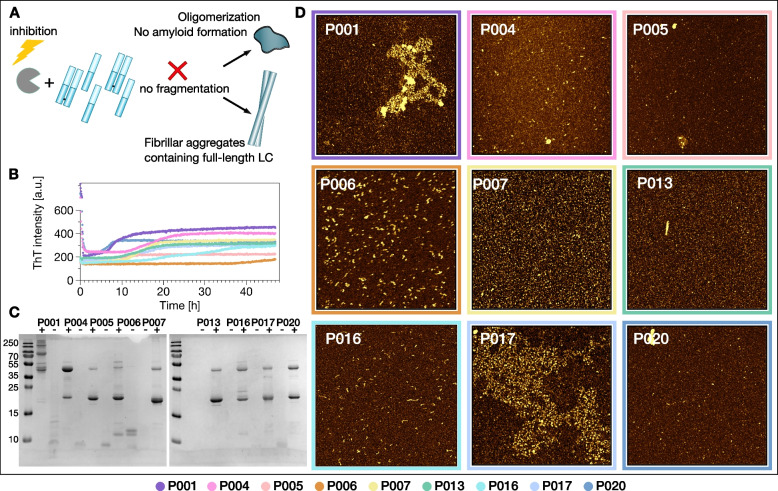
The effect of protease inhibitors on acidification-induced amyloid fibril formation by the IgLCs. **A** Inhibition of the proteases (mainly cathepsins) prevents sequence cleavage of the IgLCs and leads to virtually no amyloid fibril formation. **B** ThT fluorescence aggregation assay of the IgLC samples (35 μM monomer concentration) at pH 3 in the presence of 10 μM pepstatin A and 10 μM E-64 measured in a high-binding surface plate at 37°C (average of two repeats). **C** The light chains were analysed by non-reducing SDS-PAGE after incubation for 50 h with (+) and without (−) inhibitors. **D** AFM height images of the products of the aggregation assay after 50 h. The image size is 2 × 2 μm. The colour range represents the height from −1 to ~5 nm

The IgLCs were incubated at pH 3 in the presence of 10 μM pepstatin A and 10 μM E-64 in a high-binding plate and the potential aggregation was followed by the increase of ThT fluorescence intensity (Fig. 6). With the exception of P005 and P006, all samples showed an increase in ThT-signal. However, the ThT intensity was found to increase only by a factor of approximately two, which is almost negligible compared to the increase observed in the absence of protease inhibitors. AFM imaging of the samples displayed small, globular oligomeric structures, very different from the mature aggregates observed to form in the absence of the protease inhibitors. P006 and P016, which, based on ThT fluorescence intensity, did not or only slowly aggregate, showed slightly elongated aggregates in AFM images, which could be pre-fibrillar structures. While the incubation of the samples at pH 3 led to a complete fragmentation of the native IgLCs, the presence of the inhibitors was found to maintain the IgLCs in their original size, even after incubation for 50 h (Fig. 6C).

Finally, we tested whether the fibrils which are formed in the absence of protease inhibitors can induce the aggregation of the intact light chain. Therefore we incubated the LC proteins at 55°C, where the IgLCs are partly to fully unfolded (Additional file 2: Fig. S1) and added seeds which were produced at pH 3 or pH 4 (Additional file 3: Fig. S4). For P006 and P017, which showed both a high refolding probability upon cooling, we indeed observed an increase in ThT fluorescence intensity, which was not observed in unseeded samples, even though we could not confirm the presence of amyloid fibrils by AFM imaging. Furthermore, we pre-incubated two LC proteins, P006 and P020 as representatives, for 1 h at pH 3, the time span which lead to a complete fragmentation of the native IgLCs, and subsequently adjusted the pH to 7.4. The purpose of this experiment was to find out whether the proteolytic fragments displayed amyloidogenic potential also at neutral pH. Without pre-incubation, the IgLCs remain very stable at neutral pH and do not demonstrate noticeable aggregation, but after exposure to conditions where rapid fragmented occurs, we observed an increase in ThT fluorescence (see Additional file 3: Fig. S5). This aggregation was very rapid, in particular for P020, suggesting that the amyloidogenicity of proteolytic fragments can be very high even at neutral pH.

**Fig. 7:**
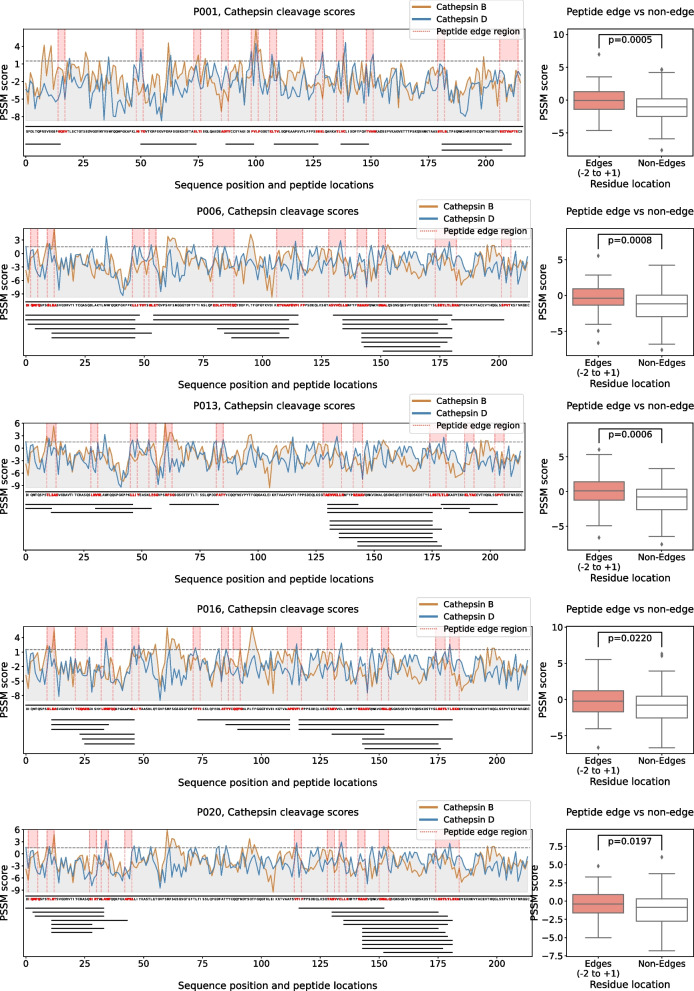
The IgLC sequences P001, P006, P013, P016 and P020 were analysed with respect to the position-dependent probability that they can be cleaved by the cathepsins B and D (brown and blue profiles, see ‘Methods’ section for details). The sequences are then aligned with the peptide fragments actually observed in the proteomics analysis of the purified and dissolved fibrils (black lines below the sequences). The PSSM scores of the sequence regions proximal to where cleavage is observed (red bands) are in all cases significantly larger than the average PSSM scores of the sequences, see Mann–Whitney test on the right-hand side. The horizontal dashed line corresponds to a PSSM score of 1.5, corresponding to a cleavage probability of approximately 81.7%

For some of our samples (P001, P006, P013, P016, P020), we used a top-down proteomics approach to determine the sequence of LC fragments that were incorporated into the amyloid fibrils formed at pH 3 and pH 4 (Fig. 7). We found different numbers of fragments, and both the constant and variable domains to be represented. The central regions of the IgLCs were almost never found to be part of the fibrils. In our fibrils formed in vitro, we only detected a fragment corresponding to most of the variable domain in P006 (pH 3), in addition to shorter fragments of the same region. We could find many similar C-terminal fragments. If we compare the fragments with potential cleavage sites of cathepsin B and cathepsin D (Fig. 7), we find that the PSSM score is significantly higher (*p*=0.0005) for the peptide edge compared to non-edge in all cases. Taken together, our inhibition and proteomics analysis strongly suggest that proteolysis by cathepsins is the key event that allows amyloid fibril formation of our IgLCs in vitro at acidic pH.

### Sequence analysis and comparison with outputs of bioinformatic prediction algorithms

The finding that all our investigated samples are able to form amyloid fibrils is even more remarkable, as none of our IgLCs appear to form amyloid fibrils in the patients. In order to understand this discrepancy, we investigated the amino acid sequences of the 9 samples characterised in this work and, in addition, the sequence of the single IgLC from an amyloidosis patient (P011) in our previous data set [13]. The sequences of the IgLCs were determined by a combination of top-down and bottom-up proteomics with specific data analysis for patient-derived IgLCs [37].

An alignment of the full-length amino acid sequences of the two λ light chains P001 and P011 and of the eight κ light chains P004, P005, P006, P007, P013, P016, P017 and P020 is presented in Additional file 1: Fig. S1 and Additional file 1: Fig. S2. The constant regions of the κ light chains are identical, apart from P013, which has a valine mutated to a leucine. An alignment of only the variable regions can be found in Fig. 1.

Besides the displayed sequence, we detected an additional IgLC proteoform in two samples (P004 and P017), and we were able to determine the sequence of the second isoform in P004, which contains two homodimers and one heterodimer, while P017 contains only one homodimer and one heterodimer at detectable concentrations. In the sample P011, which stems from the only confirmed amyloidosis patient in our cohort, we detected two other proteins with a similar mass of 20.9 and 21.1 kDa at high abundance in addition to the light chain. This contamination prevented a detailed and accurate biophysical analysis of this sample. Therefore, we excluded P011 from the further experiments and analysed solely its amino acid sequence. However, in our previous study, we could demonstrate that P011 also formed amyloid fibrils in vitro at pH 4 [13]. We used the international immunogenetics information system (IMGT) to search for the germline sequences of the investigated light chains. Both lambda IgLCs have a different origin; P001 from IGLV2-11 and P011 from IGLV1-40, which explains the large differences between the sequences (Fig. 1A, Additional file 1: Fig. S2).

The κ IgLCs originate from four different germline sequences. P005 from IGKV1-39, P004 from IGKV3-20, P007, P013 and P020 from IGKV1-5 and P006, P016 and P017 from IGKV1-33 (Fig. 1B). All of these germline sequences can be found among patients with systemic and localised amyloidosis, though IGLV2-11 is only very rarely found [31]. The secondary structure of light chains is dominated by β-strands. In order to be able to judge a possible influence of the mutations on the native structures, we modelled the three-dimensional structures of the variable regions as described in the ‘Methods’ section (Fig. 1D, E). The somatic mutations observed in most of the LCs (P006, P007, P013, P016, P017 and P020) are mainly in loop regions, and potentially have a minor impact on the structures. Between 50 and 60% of their mutations are located in the CDRs. P004 and P005 present several changes in β-strand regions, thus potentially have a stronger impact on the stability of the protein. Interestingly, mutations to proline at position 40 are observed for P013 and P006, in a region at the interface between light and heavy chain and that has been described as involved in the packing of the two chains [47]. For some of our samples (P001, P006, P013, P016, P020), we were able to use a top-down proteomics approach to determine the sequences of the fragments that were incorporated into, or interacted with, the amyloid fibrils (Fig. 7). We centrifuged the fibrils, took away the supernatant and washed the pellet several times with buffer, followed by dissolution of the fibrils with urea and mass spectrometric analysis (see ‘Methods’ section for details). This means that we detect not only fragments that are part of the fibril core, but the analysis could also include fragments that may interact very tightly with the fibril surface.

We found different numbers of fragments, and both the constant and variable domains to be represented. The central regions of the IgLCs were almost never found to be part of the fibrils. It is interesting to compare these results with the structures of IgLC amyloid fibrils extracted from patients that have recently been solved by cryo-EM. Fibrils of a λ1 AL IgLC from an explanted heart were found to consist of a 91-residue fragment of the variable domain [48], whereas in another fibril structure, a slightly shorter fragment of 77 amino acids of the variable domain was found to form the fibril core [49] In our fibrils formed in vitro, we only detected a fragment comprising almost the entire variable domain in P006 (pH 3), in addition to shorter fragments of the same region. We could find many similar C-terminal fragments, which is in accordance with the heterogeneous fragmentation positions reported recently [50]. The fragments we have detected are mostly rather short with an average molecular weight of 3.8~kDa. Previous studies already demonstrated that short peptides of 12 residues of the variable and constant domain of an amyloidogenic λ IgLC can form fibrillar structures in vitro [51]. It has been proposed that at least some proteolytic cleavage events happen after the assembly into amyloid fibrils in vivo [50, 52]. This is likely also to be the case for our amyloid fibrils assembled in vitro. However, our demonstration that some degree of proteolytic cleavage is required to initiate the formation of amyloid fibrils has also been reported for IgLCs from amyloidosis patients [53, 54] and is therefore not restricted to IgLCs from MM patients.

A previous study applied computational predictions using ZipperDB to identify so called steric zippers that drive the assembly of amyloid fibrils in IgLCs [11]. Our determination of the amino acid sequences enabled us to apply various bioinformatic prediction tools with the aim to rationalise the universal ability of our samples to form amyloid fibrils.

We tested the general amyloid propensity of the sequences with several freely available and commonly used computational algorithms such as ZipperDB [55], Waltz [56], Tango [57] and Pasta [58] (see Additional file 3 : Fig. S6, Additional file 4: Fig. S1, Additional file 4: Fig. S2, Additional file 4: Fig. S3, Additional file 4: Fig. S4 and Additional file 4: Fig. S5). Tango revealed the largest differences within the sequences, whereas Pasta displayed no amyloid potential in the variable domains. Furthermore, we also found that the sequence regions identified to be part of the fibrils do not systematically align with the ‘hot spots’ of aggregation propensity and amyloidogenicity predicted by the various algorithms (Additional file 4: Fig. S1).

In addition to our set of sequences, we also determined the aggregation propensities of a range of κ and λ sequences from different germlines selected from AL-Base [59] as a representative collection. The sequences were categorised depending on whether the patient was suffering from AL amyloidosis or multiple myeloma. The tested algorithms did not identify any clear distinctions between amyloidogenic and non-amyloid forming light chains (see Additional file 4: Fig. S6), which is in accordance with a recent analysis, which could also not identify a significant difference in the amyloid propensity between amyloidogenic and non-amyloidogenic IgLCs using Tango and Waltz [60]. The need for improved prediction algorithms for the specific case of IgLCs is increasingly recognised and has led to the recent development of two new tools, LICTOR [61] and V_L_AmY-Pred [61]. Both of these approaches are machine learning (ML) based and were trained with data sets drawn from the Boston University AL-Base, in which the IgLC sequences of amyloidosis and multiple myeloma patients, and healthy subjects are collected. The development of LICTOR was restricted to λ IgLCs, due to the higher frequency of λ-LCs in amyloidosis patients. We tested the two λ-LCs of our set with LICTOR and found that both are predicted to be non-amyloidogenic, despite P011 actually stemming from a confirmed amyloidosis patient. V_L_AmY-Pred allows also κ IgLCs to be tested and therefore enabled a more extensive application to our sequences.

Two of our IgLCs, P006 and P017, are classified by V_L_AmY-Pred as amyloidogenic with a prediction probability of 0.629 and 0.82. Both LCs originate from the germline sequence IGKV1-33, which was also found with a relatively high frequency of 18.8% among the analysed κ IgLC samples at the Mayo Clinic [31]. Similarly to the prediction by LICTOR, both of our λ IgLCs are classified as non-amyloidogenic by V_L_AmY-Pred. However, the prediction probability was rather low with 0.569 compared to an average prediction probability of 0.81. An analysis of different sequence characteristics, V_L_AmY-Pred is based on, can be found in Additional file 4: Fig. S6. Overall, applied to our set of sequences, V_L_AmY-Pred could predict the in vivo amyloid formation of 72.7% correctly, compared to 79.7% in the original test data set.

## Discussion

Various biochemical and biophysical properties, such as thermodynamic stability [9, 19, 62–64], propensity to form dimers [20, 65], ability to refold [9] or proteolytic digestibility as a proxy for protein dynamics [14] have been proposed to correlate with the different types of aggregate formation by IgLCs. In this study, we have performed an in-depth characterisation of these and additional factors for 9 out of 10 patient-derived IgLCs of which we have solved the amino acid sequences [37]. A complete overview over the data can be found in Table 1. None of these 9 samples stems from an amyloidosis patient, and yet the aforementioned biophysical and biochemical properties of dimerisation (0.1–1), thermal stability (50–64°C), efficiency of refolding (0–0.7) and trypsin digestability (0.1–1) span the full range of these parameters. This finding renders it unlikely that any of the mono-causal explanations prevalent in the current literature are able to capture the essence of IgLC aggregation in vivo. We also characterised the overall aggregation behaviour, induced by heat and low pH, in great detail. Our ThAgg-Fip approach yields a unique thermodynamic and aggregation fingerprint of each of the IgLCs of our study.

While individual patient-derived IgLCs may appear similar if characterised only in a single dimension, such as thermal stability or proteolytic digestibility, a multiparametric investigation, such as the one we present here, highlights the uniqueness of each IgLC. However, despite this uniqueness in overall behaviour, we made the remarkable discovery that every one of the IgLCs of our study can form amyloid fibrils under acidic pH conditions, as confirmed by ThT fluorescence and AFM. Mildly acidic pH values can be encountered by IgLCs in the kidney due to lysosomal proteolysis [66] and a decreased pH with a progressive chronic kidney disease (CKD) [67]. Low pH values have also previously been reported to facilitate IgLC aggregation [9, 68]. An in vitro study using human mesangial cells already suspected the importance of lysosomal processing in the process of amyloid formation [69, 70]. We were able to identify the presence of proteases, most notably cathepsins, as decisive for amyloid fibril formation in vitro because of their ability to fragment the full-length protein under acidic conditions. As additional support for this hypothesis, we were also able to show that cleavage could be prevented by the addition of cathepsin inhibitors and that the predicted cleavage sites of the cathepsins B and D align well with the observed fragments (Fig. 7). While IgLCs are unlikely to encounter pH 3 or even pH 4 in vivo*,* the presence of cathepsins probably leads to slow proteolysis even at less acidic pH (5 or 6), conditions that are physiologically relevant. The slow rate of proteolysis under such physiologically relevant conditions is not easily amenable to in vitro experiments but presumably can still be relevant for the time scale over which disease develops.

**Fig. 8:**
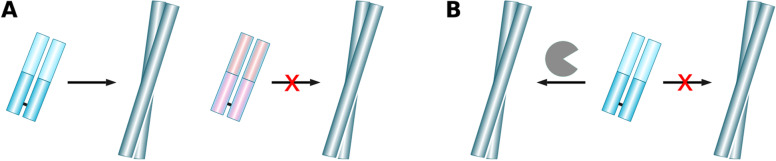
**A** Current view that there are intrinsically amyloidogenic IgLCs (blue) and non-amyloidogenic IgLCs (red). **B** Our results demonstrate that many IgLCs sequences have the potential to convert into amyloid fibrils, and it is likely additional, external factors, such as the presence of proteases under appropriate conditions, that define whether or not amyloid fibrils are formed

The availability of the full sequence information allowed us to run the sequences through a series of commonly used prediction algorithms, both older and very recent versions. Even though both LICTOR and V_L_AmY-Pred represent significant improvements over previously available prediction algorithms, they feature false negatives (LICTOR) and both false negatives and positives (V_L_AmY-Pred) in the case of our sequences. Given that both models were trained on a very large number of sequences, this shortcoming is strong evidence for the complexity of IgLC aggregation and supports our hypothesis that the origin of the differential amyloidogenicity of IgLCs in patients is not fully determined by their amino acid sequence. Our main discovery can therefore be summarised as follows (Fig. 8A, B): Many, and perhaps all, immunoglobulin light chain sequences have a significant intrinsic amyloidogenic potential, and the reason that only some are able to realise this potential is to be found in the complex interplay between intrinsic physical properties of the peptide and environmental conditions in vivo.

In particular, the presence and action of proteases such as cathepsins can facilitate the deposition of the IgLCs in the form of amyloid fibrils. Our results corroborate and provide additional rationale for, a recently presented preventative strategy against IgLC amyloid fibril formation, namely stabilisation of the folded state [71]. Our findings might also hint towards a possible mechanism for the preferential deposition of light chains in specific organs that is often observed. The biochemical micro-environment conducive to IgLC cleavage is likely to differ both between patients, as well as in different organs within a given patient. It is also important to stress that it may not be necessary to cleave the light chain quantitatively in order to allow the conversion into amyloid fibrils. In our in vitro experiments, we provided optimal conditions for efficient proteolytic cleavage, leading to relatively short sequence fragments (see Fig. 7) which are able to form amyloid fibrils particularly readily. We have provided evidence in our study that full-length IgLCs can be seeded in some cases with fibrils formed under conditions that favour cleavage (see Additional file 3: Fig. S4). While this seeding appears to be inefficient, it may still be relevant over clinical time scales, similarly to proteolysis at only mildly acidic pH. Moreover, we demonstrated that fragments formed under acidic conditions can aggregate at physiological pH conditions (see Additional file 3: Fig. S5). These results suggest that the site of fragmentation of IgLCs does not necessarily correspond to the site of aggregation and deposition.

Our results, therefore, open up new avenues towards a better understanding of AL amyloidosis. However, the more general question about the determinants of the absolute IgLC solubility in vivo still remains unanswered. Some over-produced IgLCs are very highly soluble and do not cause any damage even if present at gram scale in the patient’s plasma and urine, while others are highly insoluble and can form deposits, which among other things, can cause severe kidney damage. The observed behaviour is likely to be determined by a combination of intrinsic aggregation propensity and the concentration of the IgLC itself and of other relevant compounds.

In a previous study, we correlated the severity of kidney damage of the patients (see Additional file 1: Table S1 for a table with patient characteristics), with the biophysical and biochemical characteristics of the IgLCs [13]. Using simple pair-wise correlations between a small number of parameters, we were unable to find any clear relationship. In the present study, we employed ThAgg-fingerprinting and find that apart from the common amyloidogenicity, every IgLC has a unique fingerprint. Our results, which show a large spread of the values of the individual parameters, render it very unlikely that a single biophysical or biochemical parameter can be found to correlate with the complex in vivo solubility behaviour of IgLCs. The large spread of our samples in all crucial characteristics is ever more surprising, given the inherent bias in our data set: all proteins come from urine samples that contained a high concentration of relatively pure IgLC. Nevertheless, the degree of kidney damage they are able to induce differs significantly (see Additional file 1: Table S1). The multidimensional nature of this problem requires a multiparametric approach, such as the ThAgg-Fip method we present here. ThAgg-Fip is the result of a careful analysis of the existing literature and corresponds to a comprehensive set of the key properties that needs to be measured for a given IgLC: dimerisation, digestibility, thermal and chemical stability and temperature and concentration dependence of aggregation. The mutually contradictory results available in the literature are probably due to small sample sizes and a lack of standardisation in the biochemical and biophysical characterisation of IgLCs. Given the enormous sequence space of IgLCs to be explored, standardisation is urgently needed and we believe that ThAgg-Fip is a valuable comprehensive and standardised procedure amenable to high throughput.

In Additional file 5: Fig. S1, Additional file 5: Fig. S2 and Additional file 5: Fig. S3, we show full correlation matrices (Pearson and Spearman) and a scatter matrix between the full set of biophysical and biochemical protein properties and the clinical parameters of the patients. This relatively simple analysis already reveals a plethora of potential links between seemingly disconnected parameters. The potential of this approach is further exemplified by Additional file 5: Fig. S4, where the clinical GFR-CKD-EPI parameter, a measure for kidney function, is predicted by a linear model using the three parameters trypsin digestibility, ΔG, and *d T*_agg_/*d* log(c), with a Pearson correlation of 0.91.

While such preliminary correlations are intriguing, they should only be interpreted with care. The low amount of data points did not allow for independent data to validate the model, which makes it difficult to control for over-fitting. Also, the large number (>10) of mostly orthogonal protein properties we have been able to measure for each of our IgLCs renders such an analysis prone to occurrences of Freedman’s paradox [72] and hence necessitates a sample size at least 5–10 times larger than the one we have been able to assemble for the present study. We are confident, however, that ThAgg-Fip is easily applicable at such a scale to patient-derived samples. Combined with a maximal amount of patient clinical information (disease severity, proteomics data), the type of statistical analysis we outline here has the potential to finally unravel the origin and mechanisms of IgLC solubility and associated protein aggregation and deposition diseases.

## Conclusions

Using a multiparametric approach, such as the ThAgg-Fip method, we present here, we demonstrate that in vivo aggregation behaviour is unlikely to be mechanistically linked to any single biophysical or biochemical parameter. This multiparametric investigation highlights the uniqueness of each IgLC, even though individual IgLCs may reveal similarities if characterised only in a single dimension.

Furthermore, we show that many, and perhaps all, IgLC sequences possess an intrinsic amyloidogenic potential and the complex interplay with environmental conditions, as well as presence and action of proteases determine the ability to form amyloid depositions in vivo.

Our experiments suggest that the site of fragmentation of IgLCs might not necessary be the site of aggregation and deposition in a patient and that aggregates formed by fragments can seed the full-length IgLC.

## Methods

### Ethics, consent and permissions

The study described in this manuscript is an extension of previous work [13] and has been reviewed and approved by the ethics committee of the University Hospital Düsseldorf. All patients of whom samples were used in the study have signed an informed consent (study number 5926R and registration ID 20170664320).

### Patient-derived samples

Protein isolated from 24-h urine samples of 10~patients (5 females, 5 males, median age 64.5 years with a range between 45 and 72 years, 2 patients with a lambda isotype light chain and 8 patients with a kappa isotype chain) with MM and one patient with amyloidosis as detailed in Additional file 1: Table S1.

A histopathological examination of the patient’s kidney was not available since the corresponding invasive diagnostic procedure was not necessary for the therapy decision-making process as they were diagnosed according to IMWG criteria. These samples represent a sub-set of samples of a previous study [13] (the sample nomenclature is the same), and they were selected, because we had previously been able to determine their amino acid sequences [37] and they contained the light chain protein at sufficiently high quantity to perform the detailed biochemical and biophysical experiments and to determine the protein sequence. The protein of the patient with amyloidosis was only sequenced and not subjected to ThAgg-Fip analysis, due to lack of purity.

### Protein sample preparation

The IgLCs were purified as described previously [13]. Briefly summarised, the protein content of a 24-h urine collection was precipitated by ammonium sulfate (70% saturation) and the LCs were purified after dialysis by size exclusion chromatography on an ÄKTA pure chromatography system (GE Healthcare) using a Superdex 75 10/300 GL column and 30 mM Tris-HCl, pH 7.4, as an elution buffer. The displayed SEC chromatograms (Additional file 1: Fig. S4) are an example of one run per sample. To purify a sufficient amount of protein, a number of SEC experiments were conducted successively and the relevant fractions were combined afterwards. The chromatograms were very reproducible and the same indicated fractions were used from each individual run.

IgLC concentration was determined by measuring UV absorption at ~280 nm (extinction coefficient of 33,265 (P001), 27,640 (P004), 26,150 (P005, P006, P007, P016, P017), 33,140 (P013) and 31,650 M^−1^ cm^−1^ (P020). Pepstatin A and E-64 were dissolved in DMSO to prepare a stock solution of 1 mM, respectively.

### LC sequence characterisation and protease identification

All LC sequences were determined using a dedicated mass spectrometry workflow described in [37]. This workflow is based on the combination of bottom-up and top-down proteomics experiments with appropriate data analysis. It is important to note that the sample numbers are not identical between the present manuscript and the manuscript with the sequencing results [37], but the latter contains a correspondence table.

Briefly, light chain samples were solubilised in 8 M urea, then reduced (5 mM TCEP, 30 min) and alkylated (10 mM iodoacetamide, 30 min in the dark). After urea dilution, digestion was carried out for 3 h at 37°C (1:20) and was stopped by adding 5% FA. Peptides were desalted on Sep-Pak C18 SPE cartridge. Digests were analysed in LC-MS/MS on a Q-Exactive Plus. Peptide elution was performed with a linear gradient on a 50-cm C18 column. Mass spectra were acquired with a Top10 data-dependent acquisition mode using classical values except for the number of MS/MS μscans that was set to 4. All raw files were searched with MaxQuant against the Uniprot Homo sapiens reference proteome (74,830 entries) concatenated with the corresponding light chain sequence using trypsin as specific enzyme with a maximum of 4 miscleavages. Possible modifications included carbamidomethylation (Cys, fixed), oxidation (Met, variable) and Nter acetylation (variable). One unique peptide to the protein group was required for the protein identification. A false discovery rate cut-off of 1% was applied at the peptide and protein levels.

### Determination of the dimeric fraction

The ratios of dimers and monomers of the various IgLCs were determined by both top-down mass spectrometry (analysis of intact proteins) and analytical ultracentrifugation (AUC).

The light chains were purified by size exclusion chromatography (SEC), aliquoted, frozen and thawed before the AUC measurement. Prior to the MS measurements, the samples were dialysed against ammonium bicarbonate after the SEC and freeze-dried. The chromatograms of the different samples with the fractions used for the experiments highlighted can be found in Additional file 1: Fig. S4. All experiments of the current study were conducted with specific fractions in order to standardise the preparation since the peaks are not always symmetrical. Therefore, a bias of the measured dimer fraction due to differences in sampling cannot be fully excluded.

Sedimentation velocity (SV) experiments were performed in a XL-A ProteomeLab ultracentrifuge equipped with absorbance optics. Experiments were conducted in An-60-Ti-rotor at 20°C using a rotor speed of 60,000 rpm.

Solutions of 35 μM of IgLC samples were investigated and the radial scans were acquired at a wavelength which ensures an optimal resolution, at an absorbance of ca. 1 OD. Scans have a radial step size of 0.002 cm, i.e. radial resolution of 500 data points/cm. The sedimentation boundaries were analysed with SEDFIT (version 16p35) [73].

The *c*(*s*) model for sedimentation coefficient distributions as solutions to the Lamm equation implemented in SEDFIT was applied to the data. This model provides an approximation of a weight-average frictional ratio *f*/*f*_0_ for all particles of a distribution and based on which sedimentation coefficients can be translated into diffusion coefficients and/or molecular mass. An extension to the two-dimensional sedimentation coefficient distribution can be achieved with the *c*(*s*, *f*/*f*_0_) model, providing weight-average frictional ratios for each sedimentation coefficient of the distribution with colour-coded signal intensities [74].

During aggregation, we observed a loss of signal due to sedimentation of large assemblies during acceleration of the centrifuge to final speed for SV analysis. Absorbance was first measured at 3000 rpm to allow for quantification of the loss of material to 60,000 rpm. The wavelength for detection was varied to optimise the signal for SV analyses. The final *c*(*s*) curves were adjusted to have signals representative for the concentration left at the first time point of data acquisition.

The AUC experiments were conducted as single measurements, if the run did not indicate any disturbances. In preliminary experiments that are not shown in this manuscript, we had investigated whether the protein concentration, different buffer systems and incubation time have an influence on the *c*(*s*)-distribution. These preliminary experiments showed a high reproducibility and therefore we did not include technical replicates. The data was fitted on average seven times by using different algorithms (Marquardt-Levenberg or Simplex).

The fraction of dimer measured in relation to the overall amount of native light chains (monomer and dimer) investigated by the AUC measurements was compared to the results of mass spectrometry (MS) and previously reported results from non-reducing SDS-PAGE [13] (Fig. 2).

### Combined differential scanning fluorimetry (DSF) and dynamic light scattering (DLS) experiments

The thermal unfolding experiments of the IgLC samples as a function of protein and denaturant concentration were performed with a Prometheus Panta instrument (Nanotemper, Munich, Germany). This is a microcapillary-based (10 μl sample per capillary) instrument that allows to measure up to 48 samples in parallel. Intrinsic fluorescence can be excited at 280 nm and emission is monitored at 330 and 350 nm. The DLS experiments are performed on the same sample with a laser at 405 nm. Furthermore, the instrument also allows to measure the sample turbidity with back reflection optics.

The experiments where the initial increase and subsequent decrease in turbidity of the samples was followed (‘Bence-Jones test’, BJT) were performed by scanning the temperature from 25 to 90°C at a scan rate of 2.5°C/min. We performed these types of experiments at different concentrations for each sample and noticed a slight concentration dependence, i.e. the point of maximal turbidity shifted in most cases to lower temperatures as the protein concentration was increased. Here we report only the values at 27 μM for all samples. At the lowest concentrations measured (ca. 10 μM), the turbidity increase became in some cases too weak to be reliably quantified. However, the samples that did not show a significant turbidity increase at 27 μM also did not show any turbidity at higher concentration.

The other thermal ramping experiments were performed by scanning from 20°C (urea dependence) or 25°C (concentration dependence) to 70°C at a scan rate of 1°C/min. For the melting scans, we prepared stock solutions of the IgLCs (97 μM (P001), 150 μM (P004), 91 μM (P005), 139 μM (P006), 103 μM (P007), 97 μM (P013), 99 μM (P016), 158 μM (P017) and 81 μM (P020)). These stock solutions were filtered through a 220-nm pore size syringe filter before concentration determination and the DSF-DLS experiments. The BJT was performed at a uniform sample concentration of 30 μM and at pH 5.0, which was established by adding 100 mM Na-acetate buffer to the solutions in 30 mM Tris buffer pH 7.4. A pH value of 5 was used in the BJT as this corresponds to the original protocol used to test patient urine for BJ proteins [41].

For the concentration-dependent measurements, the stock solutions were diluted 3 times by a factor of two with 30 mM Tris buffer pH 7.4, to yield 4 different concentrations per protein, allowing to define the concentration dependence of the unfolding and aggregation temperatures. For the urea-dependent experiments, the stock solutions were diluted 5-fold into solutions of appropriate urea concentration. The final urea concentrations were 0, 0.67, 1.34, 2.01, 2.68, 3.35, 4.02, 4.69 and 5.36 M and the buffer concentration was in all cases 30 mM Tris buffer. From our initial tests, we found that the technical replicates of these experiments with the Panta instrument were very reproducible, with temperatures of unfolding and onset of turbidity usually within 0.1–0.2°C for technical replicates. We therefore did not routinely perform technical replicates of these experiments. The data reported in Table 1 stems from individual experiments.

The data was visualised as the ratio of the intrinsic fluorescence emission intensity at 350 nm over the intensity at 330 nm. For the thermal unfolding at the different protein concentrations, the melting temperature (*T*_m_) and the temperature of aggregation onset (*T*_agg_), as well as the cumulant radius, were automatically determined by the instrument software. The melting temperature corresponds to the maximum of the first derivative of the change in fluorescence intensity emission ratio. The onset of aggregation is defined by fitting the cumulant radius as a function of temperature by both a linear function (i.e. an extrapolation of the baseline) and by a sigmoidal model. The onset of aggregation is defined as the temperature at which the two fits first differ by more than 0.5%.

For the experiments in the absence of denaturant, the buffer viscosity and its temperature dependence was set to that of water. In the samples with urea, we did not correct the viscosity, which was not necessary as we did not analyse the sizes precisely. In these experiments, DLS was merely used to determine which samples had formed aggregates and should therefore be excluded from the thermodynamic analysis.

In Table 1, we report the values for *T*_m_ (pH 5 and 7.4) and *T*_agg_ (pH 7.4), as well as the concentration dependencies, *d T*_m_/*d* log(c) and *d T*_agg_/*d* log(c) (pH 7.4). We use the logarithmic derivatives, because the dependency is approximately linear on a logarithmic concentration scale, which allows to use the slope of a linear fit as a single key parameter across the entire concentration range explored. It has been shown that the determination of the unfolding temperature from fluorescence intensity ratios can lead to systematic deviations, depending on the relative intensities of the fluorescence spectra of the folded and unfolded states [75]. The use of the derivative introduced above eliminates such systematic errors, because a constant offset of all melting temperatures does not affect their dependence on protein concentration.

We also visualise the evolution of the size distribution of the sample as a function of temperature with contour plots on a logarithmic size scale. The full sets of raw data of these experiments can be found in Additional file 2: Fig. S1 and S2. In these plots, each time/temperature point corresponds to a full particle size distribution determined from a multi-species fit to the intensity autocorrelation function. These plots are automatically generated by the control software of the Prometheus Panta instrument.

For the combined chemical and thermal unfolding experiments, the data set of each protein was globally fitted to the thermodynamic two-state model recently presented [40]. The global fits are shown in Additional file 2: Fig. S3. In order to reduce the influence of aggregation on the fits, only samples containing urea were included in the fit, as the simultaneous DLS measurements had shown aggregation mostly in the absence of urea. For P004, all samples below 2 M urea were excluded on this rationale. From the global fit over all temperatures, we then determine the stability of the IgLC, Δ*G*, at 37°C. We find a significant correlation between ΔG and the m-value, i.e. *m*=*d* Δ*G*/*d* [urea] [76]. We therefore fix the m-value to a common value for all different light chains and focus on the resulting differences between ΔG. Error estimates of the obtained values were obtained by 100-fold bootstrapping by resampling the different capillaries with replacement.

### Measurement of aggregation kinetics

Different solution conditions (acidic pH values) were tested for their potential to induce aggregation of patient-derived, purified IgLCs. In order to prepare the samples at different acidic pH values (pH 2, pH 3, pH 4), protein solutions of different concentrations were diluted from 30 mM Tris-HCl pH 7.4 1:1 into 300 mM citric acid buffer at the desired pH value. Two or three replicates of each solution were then pipetted into a high-binding surface plate (Corning #3601, Corning, NY, USA). The aggregation kinetics were monitored in the presence and absence of small glass beads (SiLibeads Typ M, 3.0 mm). The plates were sealed using SealPlate film (Sigma-Aldrich #Z369667). The kinetics of amyloid fibril formation were monitored at 37°C either under continuous shaking (600 rpm) or under quiescent conditions by measuring ThT fluorescence intensity through the bottom of the plate using a FLUOstar (BMG LABTECH, Germany) microplate reader (readings were taken every 150 or 300 s). In order to compare the factor of the increase of the ThT fluorescence emission intensity between the samples, the ThT fluorescence emission intensity at the end of the experiment was compared with the lowest emission value. The halftimes of the aggregation reaction are defined as the point where the ThT intensity is halfway between the initial baseline and the final plateau. The halftimes were obtained by individually fitting the curves using the following generic sigmoidal equation [77]

$$Y={y}_i+{m}_it+\left({y}_f+{m}_ft\right)/\left(1+{e}^{-\left(t-t_{50}/k\right)}\right)$$

where *Y* is the ThT fluorescence emission intensity, *t* is the time and *t*_50_ is the time when 50% of maximum ThT fluorescence intensity is reached. The initial baseline is described by *y*_*i*_+*m*_*i*_*t* and the final baseline is described by *y*_f_+*m*_f_t. While this equation does not describe the underlying molecular processes of aggregation, it does allow determination of the main phenomenological parameter, the half time, of each experiment.

The aggregation kinetics experiments were conducted by investigating 2–3 technical replicates per condition. To test the reproducibility, the experiments were repeated for selected samples. The sequence regions incorporated into the fibrils were determined from one aggregated sample in each case. At the end of the experiments, the amount of aggregated protein was determined by combining the replicates and pelleting the aggregation product for 1 h at 16,100*g* followed by measuring the soluble content by UV absorbance at 280 nm of the supernatant, and correcting for the absorbance of the ThT. In order to investigate whether the aggregation can be seeded at acidic pH, fibrils were produced by incubating 35 μM protein solution at pH 3 and pH 4 in a high-binding plate and in a 2-ml Eppendorf tube in presence of glass beads under shaking conditions at 37°C. The presence of fibrillar aggregates was confirmed by AFM. For seed generation, the fibril solutions obtained from the plate and from an Eppendorf tube were homogenised using an ultra-sonication bath Sonorex RK 100 H (Bandelin, Germany) for 300 s. The seeded aggregation experiments were performed in high-binding surface plates under quiescent conditions with 35 μM P016 monomer and the pre-formed seeds were added to a final concentration of 5% of the monomer solution at the desired pH value. The seeds were added either at the beginning or after 6 h pre-incubation of the monomer solution at 37°C.

We furthermore investigated the seeding potential at neutral pH, where the protein remains largely unaffected by proteolytic activity. We used pre-formed fibrils, which were produced at pH 3 and pH 4 in an Eppendorf tube as described above at 100 μM LC concentration. The seeds were additionally washed to remove the soluble fragments by centrifuging the sample at 137,000*g* at 20°C for 45 min and re-suspending the pellet in 150 mM citric acid. This washing procedure was carried out three times. The seeded aggregation experiments were performed in high-binding surface plates under agitation conditions with 50 μM monomer solution and seeds added to a final concentration of 5%.

### Prevention of amyloid formation at acidic pH values

In order to investigate whether the cleavage of the IgLCs at acidic pH values was responsible for the amyloid fibril formation, we tested whether inhibiting some of the identified proteases will prevent the formation of ThT-positive aggregates.

Therefore, the IgLCs (35 μM monomer concentration) were incubated as described above in a high-binding surface plate under quiescent conditions in the presence of 10 μM pepstatin A and E-64, respectively. E-64 is an irreversible and highly selective cysteine protease inhibitor, and pepstatin A is a reversible inhibitor of acidic proteases (aspartic proteases) and can be used in a mixture with other enzyme inhibitors. Further experiments were conducted with P005 and P016 in the presence of 1 μM pepstatin A or E-64 or 1 μM or 10 μM pepstatin A and E-64 under agitation conditions. The morphology of aggregates was investigated by AFM.

### Fibril fragment determination

The aggregation products formed in Eppendorf tubes under agitation conditions at pH 3 and pH 4 were centrifuged using an Optima MAX-XP ultracentrifuge (Beckman Coulter) in a TLA-55 rotor at 40,000 rpm at 20°C for 45 min. The pellet was re-suspended in 150 mM citric acid (pH 3 or pH 4) and centrifuged again for 45 min. This washing procedure to remove the soluble fragments was conducted three times. The washed aggregates were dissolved in 6 M urea and subsequently analysed by mass spectrometry.

A Dionex UltiMate 3000 RSLC Nano System coupled to an Orbitrap Fusion Lumos mass spectrometer fitted with a nano-electrospray ionisation source (Thermo Scientific) was used for all experiments. Five microliters of reduced/alkylated protein samples in solvent A were loaded at a flow rate of 5 μL min^−1^ onto an in-house packed C4 (5 μm, Reprosil) trap column (0.150 mm i.d. × 35 mm) and separated at a flow rate of 0.5 μL min^−1^ using a C4 (5 μm, Reprosil) column (0.075 mm i.d. × 28 cm). The following gradient was used: 2.0% B from 0–10 min; 20% B at 11 min; 60% B at 22 min; 99% B from 25–30 min; and 2.0% B from 30.1 to 35 min. Solvent A consisted of 98% H_2_O, 2% ACN and 0.1% FA, and solvent B 20% H_2_O, 80% ACN and 0.1% FA. MS scans were acquired at 120,000 resolving power (at m/z 400) with a scan range set to 550–2,000 m/z, four microscans (μ scans) per MS scan, an automatic gain control (AGC) target value of 5×105 and maximum injection time of 50 ms. MS/MS scans were acquired using the Data-Dependent Acquisition mode (Top 4) at 120,000 resolving power (at m/z 400) with an isolation width of 1.2 m/z, five μ scans, an AGC target value of 5×105 and maximum injection time of 250 ms. For fragmentation, electron transfer dissociation with 10 ms of reaction injection time and a supplemental higher-energy collisional dissociation with normalised collision energy (NCE) of 10% (EThcD) was used.

All data were processed with ProSightPC v4.1 (Thermo Scientific) and Proteome Discoverer v2.4 (Thermo Scientific) using the ProSightPD 3.0 node. Spectral data were first deconvoluted and deisotoped using the cRAWler algorithm. Spectra were then searched using a two-tier search tree with searches against the corresponding LC sequences. The search 1 consists of a ProSight Absolute Mass search with MS1 tolerance of 10 ppm and MS2 tolerance of 5 ppm. The search 2 is a ProSight Biomarker search with MS1 tolerance of 10 ppm and MS2 tolerance of 5 ppm. Identifications with E-values better than 1e−10 (−log (E-value) = 10) were considered as confident hits.

### Atomic force microscopy (AFM)

Atomic force microscopy height images were acquired after the aggregation kinetic measurements. Ten microliters of each sample (after diluting 1:4 with dH_2_O) were deposited onto freshly cleaved mica. After drying, the samples were washed 5 times with 100 μL of dH_2_O and dried under gentle flow of nitrogen. AFM images were obtained using a NanoScope V (Bruker) atomic force microscope equipped with a silicon cantilever ScanAsyst-Air with a tip radius of 2–12~nm. The images were analysed with the software Gwyddion 2.56 to measure height profiles and investigate a possible twisting of the fibrillar aggregates.

### Microfluidic diffusional sizing and concentration measurements

To investigate the influence of acidic pH on the samples, the samples were analysed using SDS-PAGE and FluidityOne. Fluidity One is a microfluidic diffusional sizing (MDS [42]) device, which measures the rate of diffusion of protein species under steady-state laminar flow and determines the average particle size from the overall diffusion coefficient. The protein concentration is determined by fluorescence intensity, as the protein is mixed with ortho-phthalaldehyde (OPA) after the diffusion, a compound which reacts with primary amines, producing a fluorescent compound [78]. To measure the influence of pH on the average size of the molecules in the solution, the protein was pre-incubated at acidic pH values with 150 mM citric acid (pH 3 or pH 4). The IgLC solution was incubated in an Eppendorf tube at 37°C under quiescent conditions. After different incubation times, 6 μL of the solutions were pipetted onto a disposable microfluidic chip and measured with the Fluidity One (F1, Fluidic Analytics, Cambridge, UK). The samples were also analysed by SDS-PAGE, according to a previously published protocol [13].

### Modelling and data and sequence analysis

The sequences of the IgLC samples of this study were parsed with IMGT, the international ImMunoGeneTics information system and were aligned in order to investigate the amino acid changes between the germline sequences and the sequences under study. The sequences were also analysed using different online bioinformatic tools (ZipperDB, Tango, Pasta, CamSol), which have been developed to predict the aggregation propensity/solubility of proteins based on their amino acid sequences. All the light chain sequences were modelled using the Lyra software [79], and insertions and deletions were refined using the modeller [80] software using default parameters. The structure renderings were created using PyMol [81].

All the substrates for Cathepsin B and D were retrieved from the Merops database [82]. Since limited available experimental evidence was present in the Merops database on the other proteases, they were removed from further analyses.

The 4 residues preceding and following the proteolytic sites were used to build two position-specific weight matrices (PSSMs) for Cathepsin B and D with the Biopython [83] motifs packages using a pseudocount of 5 and the background distribution of the human proteome. All the residues in the light chain sequences were scored using the PSSMs, and at each position a cleavage log-odds score was defined as the largest of the log-odds scores obtained for the two PSSMs. We then tested if the cleavage sites potentially produced by the proteases support the peptides experimentally identified by mass spectrometry. To do so, we identified the regions in the sequences proximal to the N- and C-terminal residues from the experimental peptide and tested if the cleavage log-odds scores within such region was significantly larger than outside. The proximal region was defined as the union of all residues that are at most two residues before a peptide’s N terminal and at most one residue after it, plus all residues that are at most one residue before a peptide’s C-terminal and at most two residues after it, in the corresponding light chain sequence. By including the residues near the observed peptide termini, we account for the potential effect of exopeptidases, and for imprecisions in the data used for training the PSSMs. The cleavage scores in the proximal regions were then compared to the scores outside of it by performing a one-tailed Mann–Whitney *U* test, and in all cases, the results were under the significance threshold of 0.05. The plots visualising these results were generated with the Python Matplotlib library.

In order to investigate the correlations between the experimental quantities measured of the IgLCs, numerical values were needed for all measurements. This necessitated an arbitrary conversion of fraction refolded by DSF to a numerical scale of 0.33, 0.50 or 0.66, for small, medium and large, respectively. Similarly, refolding at 2M urea was converted to an ordered categorical scale [0,1,2] for 0, <50 and >50, respectively. The ΔG-value was not included for P016, and m-values were not used because they were globally fitted and therefore shared between all data sets. All other non-numerical cells in Table 1 were interpreted as missing values. Before training an Elastic Net model on the data, missing values were set to the mean of the given observable and all values were normalised to the mean value with unit variance. The following limited set of parameters were chosen to eliminate the most internally correlated measurements (e.g. dimerization by three different methods): *T*_m_ DSF (pH 5), *T* (maximum turbidity at pH 5), *T*_m_ DSC, *d T*_m_/*d* log(c) (pH 7.4), *d T*_agg_/*d* log(c) (pH 7.4), cumulant radius (pH 7.4, 70°C), dimerization by AUC, fraction refold by DSC, ΔG (37°C) and digestability by trypsin. Model training was done using a grid search with 4-fold cross validation using negative mean squared error as the loss function. A separate validation set was not created because of the low number of data points. This analysis was performed in Python 3.7 using packages Numpy, Pandas, SKLearn, Matplotlib and Seaborn.

## Supplementary Information

**Additional file 1: Table 1.** Patient characteristics at the time of examination. The samples were taken at the time point of diagnosis. The patients are categorised according to their CKD stage: Group I "good" stage 1+2, *n*=5 (P004, P007, P011, P017, P020), Group II intermediate stage 3, *n*=4 (P001, P005, P013, P016) and Group III bad stage 4+5, *n*=1 (P006). Apart from P007, which presented an acute kidney injury, all patients had a chronically damaged kidney. **Figure A.1.** Full length sequence alignment of the κ light chains of our study. The framework regions (FR) are marked with blue, the complementarity determining regions (CDRs) with red. **Figure A.2.** Full length sequence alignment of the λ light chains of our study. The framework regions (FR) are marked with blue, the complementarity determining regions (CDRs) with red. **Figure A.3.** (A) Distribution of sedimentation coefficients (c(s)) of the IgLC samples of this study and (B) application of the c(s, f/f0)-model determined by sedimentation velocity experiments at 60.000 rpm. We find that the form factors of the dimers differ within the sample set. The dimers can appear very globular e.g. P020 with a f/f0 of 1.14 or elongated such as P006 f/f0 of 2.22 and P007, P013 and P017 f/f0 between 1.54 and 1.83. However the signal for the dimer of P006 is very low. (C) shows the variation of f/f0 between the dimers compared to the monomers. The monomers of P004 and P020 were excluded due to their low signal intensity. **Figure A.4.** Size exclusion chromatograms of the different IgLC samples of this study. The fractions used for the remaining experiments in this study are marked in blue.

**Additional file 2: Figure A.1.** Concentration dependent thermal unfolding experiments of the IgLC samples of our study. The samples were scanned from 25-70◦C at 1◦C per minute. In each case, the evolution of the ratio of the intrinsic fluorescence intensities at 350 and 330 nm is shown on top, and the evolution of the size distribution, measured by dynamic light scattering (DLS) is shown on a logarithmic scale on the bottom as a contour plot. Contours are spaced with 0.1 and the lowest contour drawn is at 0.03 amplitude. Each sample was measured at 4 concentrations, as the undiluted stock solution as well as 3 dilutions by a factor of 2 each. The concentrations of the stock solutions are 97 μM (P001), 150 μM (P004), 91 μM (P005), 139 μM (P006), 103 μM (P007), 97 μM (P013), 99 μM (P016), 158 μM (P017) and 81 μM (P020). **Figure A.2.** Temperature-dependent chemical denaturation of the IgLC samples of this study by urea. In each case, the evolution of the ratio of the intrinsic fluorescence intensities at 350 and 330 nm is shown on top, and the evolution of the size distribution, measured by DLS is shown on a logarithmic scale on the bottom as a contour plot. Contours are spaced with 0.1 and the lowest contour drawn is at 0.03 amplitude. The protein concentration corresponds to a 5-fold dilution of the stock solution used for the thermal denaturation in Figure A.1. The urea concentrations are in each case 0, 0.67,1.34, 2.01, 2.68, 3.35, 4.02, 4.69, 5.36 M. The samples were scanned from 20-70◦C at 1◦C per minute. **Figure A.3.** Global fits of the temperature-dependent chemical denaturation of the IgLC samples of this study by urea. The data is the same as in the previous figure, but instead of using the fluorescence intensity ratio, the fits are performed simultaneously on the fluorescence intensities at both 330 nm (shown here) and 350 nm. **Figure A.4.** Results from the global fits of the temperature-dependent chemical denaturation of the IgLC samples of this study by urea. Shown is the fraction of unfolded protein as a function of urea concentration at different temperatures. **Figure A.5.** A modern implementation of the historical test for Bence-Jones proteins. (A) Illustration of the original test for Bence-Jones proteins, i.e. free IgLCs, in urine?. After adjustment to pH 5, the urine sample is heated and turns cloudy at approximately 50◦C. If further heating to temperatures close to the boiling point leads to a decrease in turbidity, the presence of Bence-Jones proteins is confirmed. (B) Illustration of the BJT in microcapillaries, here shown in cross-section. The sample turbidity is measured with a back-reflection optics. Initially, the IgLCs are in their soluble, monomeric or dimeric form (1). Protein aggregation at intermediate temperatures leads to more scattering and less back-reflection (2), and subsequent heating leads to an increase in back-reflection (3) in the case of typical Bence-Jones behaviour. (C) Sample turbidity is measured as IgLC samples at pH 5 are heated from 25 to 90◦C at 2.5◦C/min.

**Additional file 3: Figure A.1.** AFM-height-images of aggregates prepared in different reaction vessels. We used as seeds both fibrils which had been prepared in a high-binding surface plate in the presence of glass beads (A), as well as fibrils which had been prepared in the same volume in an 2 ml Eppendorf tube with glass beads (B). The presence of fibrils was confirmed using atomic force microscopy. Although the presence of fibrils could be confirmed in both setups, the total ThT-fluorescence intensity was lower if seeds prepared in an Eppendorf tube were used. The image scale is 5 x 5 μm. The colour range represents the height from -2 to 15 nm. **Figure A.2.** Aggregation assays of P016 (35 μM monomer concentration) at (A) pH 3 and (B) pH 4 monitored in a high-binding surface plate in the presence of glass beads and conditions of mechanical agitation and with addition of glass beads after 24 h pre-incubation without shaking and the (C) aggregation halftimes (top). Aggregation assays of P016 at (D) pH 3 and (E) pH 4 in a high-binding plate under quiescent conditions. Seeds prepared in a high-binding plate and prepared in an Eppendorf tube are added at the beginning and after 6 h pre-incubation and (F) the halftimes are analysed. The pre-incubation times (24 or 6 h) are subtracted from the halftimes. **Figure A.3.** The influence of acidic pH on the IgLC samples. (A) Illustration of the finding that acidic pH enables the naturally present proteases in the IgLC samples to cleave the IgLCs into fragments, that are then subsequently forming amyloid fibrils. (B) The fraction of native protein (monomer and dimer combined) at different incubation times determined by SDS-PAGE (left: pH 2, middle: pH 3, right: pH 4) and (C) the hydrodynamic radius in nm (left: pH 3, middle: pH 4) and the normalized concentration measured by Fluidity One (right: pH 4). **Figure A.4.** (A) Aggregation experiment at 55◦C monitored in a non-binding surface plate under agitation conditions in the presence of glass beads. 5% Seeds which were produced at pH 3 or pH 4 in an Eppendorf tube were added to 50 μM light chain. (B) The soluble content was determined after the experiment by UV-absorbance. AFM-height-images of the light chains at the end of the experiments (C) and of aggregated P006 with the positive ThT-signal (D). Fibrils at pH 3 do not display any twist. The image scale is 5 x 5 μm. The colour range represents the height from -2 to 5 nm. **Figure A.5.** Aggregation experiment of (A) P006 and (B) P020. After pre-incubation at pH 3 under quiescent conditions at 37◦C, the sample was diluted with 1 M Tris-HCl, pH 11.1, to achieve a pH of 7.4 and a protein concentration of 35 μM (pink). The aggregation was monitored in a high-binding surface plate under agitation conditions in the presence of glass beads. The negative control without pre-incubation (blue) showed no increase in ThT-fluorescence over a time course of 120 h. **Figure A.6.** The sequence regions of P001, P006, P013, P016 and P020 that were found in the aggregates formed at acidic pH values and identified by mass spectrometry. The sequence regions were analysed according to their charge at the depicted pH value (blue) and hydrophobicity score (orange) using the Peptide Analyzing Tool from Thermofisher. The hydrophobicity score is based on the index proposed by Krokhin and Spicer?. The AGG parameters of the sequence fragments were analysed using the TANGO algorithm, the horizontal line indicates the AGG parameter value of the full length sequence.

**Additional file 4: Figure A.1.** The IgLC sequences P004, P005, P007, P011 and P017 were analysed with different amyloid prediction tools and the predicted aggregation hot spots visualised on the sequence. Amyloid propensity was determined with the algorithm PASTA (red pH 3, blue pH 4) and the amyloidogenic sequence regions with the algorithms TANGO and WALTZ. The sequence parts which were found in the aggregates formed at acidic pH values are marked with black lines. **Figure A.2.** (A) Intrinsic solubility score measured by CamSol and (B) aggregation parameter determined by the Tango algorithm at pH 3, pH 4 and pH 7, (C) aggregation free energy computed with the Pasta algorithm. **Figure A.3.** The κ IgLC sequences P004, P005, P006 and P007 were analysed with different amyloid prediction tools and the results visualised on the sequence. Rosetta energy was determined by ZipperDB, amyloidogenic sequence regions with the algorithms Waltz, Tango and Pasta. The analyses with Tango were performed at pH 3 (red), pH 4 (green) and pH 7 (blue). **Figure A.4.** The κ IgLC sequences P013, P016, P017 and P020 were analysed with different amyloid prediction tools and visualised on the sequence. Rosetta energy was determined by ZipperDB, amyloidogenic sequence regions with the algorithms Waltz, Tango and Pasta. The analyses with Tango were performed at pH 3 (red), pH 4 (green) and pH 7 (blue). **Figure A.5.** The λ IgLC sequences P001 and P011 were analysed with different amyloid prediction tools and visualised on the sequence. Rosetta energy was determined by ZipperDB, amyloidogenic sequence regions with the algorithms Waltz, Tango and Pasta. The analyses with Tango were performed at pH 3 (red), pH 4 (green) and pH 7 (blue). **Figure A.6.** Variable κ region sequences were analysed with Tango at pH 7 (A) and pH 3 (B). The sequences, which originate from different germline sequences were selected from AL-Base and were categorised according to whether the patient was suffering from AL-Amyloidosis or MM. IGKV1-5 (blue) AL *n*=10, MM *n*=9; IGKV1-33 (violet) AL *n*=8, MM *n*=10; IGKV1-39 (green) AL *n*=10, MM *n*=10; IGKV3-20 (orange) AL *n*=7, MM *n*=10. Variable lambda region sequences were analysed with Tango at pH 7 (C) and pH 3 (D). The sequences, which originate from different germline sequences were selected from AL-Base and were categorised according to whether the patient was suffering from AL-Amyloidosis or MM. IGLV1-40 (orange) AL *n*=10, MM *n*=5; IGLV2-11 (violet) AL *n*=2, MM *n*=11. The open circles indicate the germline sequence. The aggregation related features according to? (E) consensus hydrophopbicity of the CDRs, (F) percentage of the gatekeeper residues in FRs and (G) average IUPred score calculated for the different segments of the variable region of investigated IgLCs. The colour code is equivalent to the previous figures.

**Additional file 5: Figure A.1.** Pearson correlation matrix between all experimental parameters of this study and clinical patient descriptors. The number of data points for each separate correlation can be seen in Figure 3 below. **Figure A.2.** Spearman correlation matrix between all experimental parameters of this study and clinical patient descriptors. The number of data points for each separate correlation can be seen in Figure 3 below. **Figure A.3.** Scatter matrix that shows the pairwise correlation plots in the off-diagonal elements, and a histogram of the parameter values in the diagonal elements. **Figure A.4.** An elastic net model was trained to predict (GFR-CKD-EPI) using all the biophysical parameters measured of the light chains. a) Negative mean squared errors of the models are shown for training and test sets using 4-fold cross validation as a function of the regularisation parameters. No data points were omitted as a separate validation set because of the low amount of data. b) The best model with Alpha = 3.98 and L1Ratio = 1.0 is shown. This model uses only the three parameters trypsin digestibility, ΔG, and dTagg/dlog c, with a Pearson correlation of 0.91. The mean error of cross validation of the model is 18.97±12.14. The influence of dTagg/dlog c seems to mainly arise from a single data point, so the two other parameters are probably more robust. This type of behaviour emphasises the need for significantly larger data sets than the ones that are currently available. Searching for correlations within combinations of parameters provides a large freedom that needs to be constrained by the size of the data set.

## Data Availability

All data generated or analysed during this study are included in this published article, its supplementary information files and publicly available repositories: 10.11583/DTU.21646691.v1.

## Abbreviations

MM: Multiple myeloma
IgLC: Immunoglobulin light chain
LC: Light chain
ThAgg-Fip: Thermodynamic and Aggregation Fingerprinting
LCDD: Light chain deposition disease
CDRs: Complementarity-determining regions
MS: Mass spectrometry
AUC: Analytical ultracentrifugation
SEC: Size exclusion chromatography
DLS: Dynamic light scattering
DSC: Differential scanning calorimetry
AFM: Atomic force microscopy
E-64: Trans-Epoxysuccinyl-L-leucylamido(4-guanidino)butane
ML: Machine learning
