# Supplementary material for: Widespread amyloidogenicity potential of multiple myeloma patient-derived immunoglobulin light chains
Source: BMC Biol. 2023 Feb 3;21:21. doi: 10.1186/s12915-022-01506-w (PMC9898917; doi:10.1186/s12915-022-01506-w)
Supplement: Supplementary file 1 — Additional file 1: Table 1. Patient characteristics at the time of examination. The samples were taken at the time point of diagnosis. The patients are categorised according to their CKD stage: Group I "good" stage 1+2, n=5 (P004, P007, P011, P017, P020), Group II intermediate stage 3, n=4 (P001, P005, P013, P016) and Group III bad stage 4+5, n=1 (P006). Apart from P007, which presented an acute kidney injury, all patients had a chronically damaged kidney. Figure A.1. Full length sequence alignment of the κ light chains of our study. The framework regions (FR) are marked with blue, the complementarity determining regions (CDRs) with red. Figure A.2. Full length sequence alignment of the λ light chains of our study. The framework regions (FR) are marked with blue, the complementarity determining regions (CDRs) with red. Figure A.3. (A) Distribution of sedimentation coefficients (c(s)) of the IgLC samples of this study and (B) application of the c(s, f/f0)-model determined by sedimentation velocity experiments at 60.000 rpm. We find that the form factors of the dimers differ within the sample set. The dimers can appear very globular e.g. P020 with a f/f0 of 1.14 or elongated such as P006 f/f0 of 2.22 and P007, P013 and P017 f/f0 between 1.54 and 1.83. However the signal for the dimer of P006 is very low. (C) shows the variation of f/f0 between the dimers compared to the monomers. The monomers of P004 and P020 were excluded due to their low signal intensity. Figure A.4. Size exclusion chromatograms of the different IgLC samples of this study. The fractions used for the remaining experiments in this study are marked in blue. [file 12915_2022_1506_MOESM1_ESM.pdf]

A Additional file 1

Table 1. Patient characteristics at the time of examination. The samples were taken at the time point of diagnosis. The patients are categorised according to their CKD stage: Group I "good" stage 1+2, n=5 (P004, P007, P011, P017, P020), Group II intermediate stage 3, n=4 (P001, P005, P013, P016) and Group III bad stage 4+5, n=1 (P006). Apart from P007, which presented an acute kidney injury, all patients had a chronically damaged kidney.

Patient	Age	Gender	Disease type	Isotype	FLC Serum [mg/l]	FLC Urine [mg/l]	TPU [g/24h]	Kidney function creatinin [mg/dl]	GFR-CKD-EPI [ml/min]	CKD Stadium	Kidney function recovered? Improvement = 30%	Kidney function recovered Creatinine [mg/dl]
**P001**	47	male	MM	λ	3750	1060	13	1.3	50	3	Yes	0.9
**P004**	72	male	MM	κ	5280	7650	4	1.1	90	1	Yes	0.9
**P005**	65	female	MM	κ	1250	6140	3	1.2	48	3	n/a	acute kidney injury
**P006**	66	female	MM	κ	2460	6880	3	1.9	27	4	Yes	1.3
**P007**	54	male	MM	κ	898	n/a	1	1	83	2	No	1.3
**P011**	45	female	AL	λ	120	n/a	6.2	0.7	108	1	No	0.8
**P013**	65	male	MM	κ	11000	n/a	3	1.5	50	3	No	1.7
**P016**	59	male	MM	κ	1150	175	3	1.6	45	3	No	1.2
**P017**	72	female	MM	κ	1380	n/a	4	0.7	90	1	No	0.8
**P020**	64	female	MM	κ	4120	n/a	2.4	0.8	74	2	n/a	0.9

```
                       FR1            CDR1        FR2      CDR2
P004a     EIVLTQSPGTLSLSPGERATLSCRASQSVSSSYLAWYQQKPGQAPRLLIYDASTRATGIP 60
P004b     EIVLSQSPDTLSLSPGERATLSCRADKSVSSNYVAWYQQKPGQAPRLLIYDAFTRATGIP 60
P013      DIQMTQSPSTLSASVGDAVTITCRASQSL-NVWLAWYQQKPGKPPKLLIYEASNLESGVP 59
P020      DIQMTQSPSTLSTSVGDRVTITCRASQSI-RTWLAWYQQKPGKAPKLLIYKASTLETGVP 59
P007      DIQMTQSPSTLSASVGDRVTITCRASQSL-SSSLAWYQQKPGKAPKLLIYDASSLETGVP 59
P005      DIQMTQSPSSLSASVGDRVSITCRASESI-SSYVNWYQQKPGKAPKLLIYTASSLQSGVP 59
P006      DIQMTQSPSSLSASVGDRVTITCQASQDL-AKYLNWYQQKPGKPPKLLIYDTSNLETGVP 59
P016      DIQMTQSPSSLSASVGDRVTITCQASRDI-SNYLNWYQQKPGKAPMLLIYAASNLQTGVP 59
P017      DIQMTQSPSSLSASVGDRVTITCQASQDL-GNYLNWYQQKPGKAPRLLIYDASDLEEGVP 59
          :* ::***.:** * *: .:::*:*...:    : *******: * **** :     *:*
                         FR3              CDR3     FR4
P004a     DRFSGSGSGADFLLTISSLEPEDFAMYYCQQYGR-SPYTFGPGTKVDIKRTVAAPSVFIF 119
P004b     DRFSGSGSETDYTLTISTLEPEDFAVYYCQQYGR-SPYTFGPGTKVDIKRTVAAPSVFIF 119
P013      SRFSGSGSGTEFTLTISSLQPDDFATYYCQQYNS-YPYTFGQGAKLEIKRTVAAPSVFIF 118
P020      SRFSGSGSGTEFTLTISSLQPEDFATYYCQQYND-YSGTFGQGTKLEIKRTVAAPSVFIF 118
P007      SRFSGSGSGTEFTLSISSLQPDDFATYYCQHYNS-YSLTFGQGTKVEIKRTVAAPSVFIF 118
P005      PRFSGSASGTDFTLTISSLQPEDFATYYCQQSYS-TPITFGQGTRLEIKRTVAAPSVFIF 118
P006      SRFSN-GGGTDFTFTINSLQPEDLATYYCQQYDDF-PLTFGPGTKVDIKRTVAAPSVFIF 117
P016      SRFSGSGSGTDFTFTISSLQPEDLATYYCQQYGNL-PLTFGGGTKVEIKGTVAAPSVFIF 118
P017      SRFSGSGSGTDFTFTISSLQPEDFATYYCQQYHTLPPLTFGGGTKVDVKRSLAAPSVFIF 119
           ***. .. ::: ::*.:*:*:*:* ****:       *** *:::::* ::********

P004a     PPSDEQLKSGTASVVCLLNNFYPREAKVQWKVDNALQSGNSQESVTEQDSKDSTYSLSST 179
P004b     PPSDEQLKSGTASVVCLLNNFYPREAKVQWKVDNALQSGNSQESVTEQDSKDSTYSLSST 179
P013      PPSDEQLKSGTASVVCLLNNFYPREAKVQWKVDNALQSGNSQESVTEQDSKDSTYSLSST 178
P020      PPSDEQLKSGTASVVCLLNNFYPREAKVQWKVDNALQSGNSQESVTEQDSKDSTYSLSST 178
P007      PPSDEQLKSGTASVVCLLNNFYPREAKVQWKVDNALQSGNSQESVTEQDSKDSTYSLSST 178
P005      PPSDEQLKSGTASVVCLLNNFYPREAKVQWKVDNALQSGNSQESVTEQDSKDSTYSLSST 178
P006      PPSDEQLKSGTASVVCLLNNFYPREAKVQWKVDNALQSGNSQESVTEQDSKDSTYSLSST 177
P016      PPSDEQLKSGTASVVCLLNNFYPREAKVQWKVDNALQSGNSQESVTEQDSKDSTYSLSST 178
P017      PPSDEQLKSGTASVVCLLNNFYPREAKVQWKVDNALQSGNSQESVTEQDSKDSTYSLSST 179
          ************************************************************

P004a     LTLSKADYEKHKVYACEVTHQGLSSPVTKSFNRGEC 215
P004b     LTLSKADYEKHKVYACEVTHQGLSSPVTKSFNRGEC 215
P013      LTLSKADYEKHKLYACEVTHQGLSSPVTKSFNRGEC 214
P020      LTLSKADYEKHKVYACEVTHQGLSSPVTKSFNRGEC 214
P007      LTLSKADYEKHKVYACEVTHQGLSSPVTKSFNRGEC 214
P005      LTLSKADYEKHKVYACEVTHQGLSSPVTKSFNRGEC 214
P006      LTLSKADYEKHKVYACEVTHQGLSSPVTKSFNRGEC 213
P016      LTLSKADYEKHKVYACEVTHQGLSSPVTKSFNRGEC 214
P017      LTLSKADYEKHKVYACEVTHQGLSSPVTKSFNRGEC 215
          ************:***********************
```

Figure A.1. Full length sequence alignment of the κ light chains of our study. The framework regions (FR) are marked with blue, the complementarity determining regions (CDRs) with red.

```
                                FR1                       CDR1              FR2            CDR2
P001      GPDLTQPRSVSGSPGQSVTLSCTGTSSDVGGYNYVSWYQQHPGKAPKLMIYDVTKRPSGV 60
P011      EAPLTQPPSVSGAPGQRVTLSCTGSSSNLGAGWDVHWYQQLPGTVPKLLIYADRNRPSGV 60
             **** ****:*** *******:**::*.   * **** **..***:**   :*****
                   FR3                           CDR3       FR4
P001      PDRFSGSKSGTTASLTISGLQAEDEADYYCCSYAG-IDIFVLFGGGTKLTVLGQPKAAPS 119
P011      PERFSGSKSGTSATVAIAGLQAEDEADYYCQSYDSALSGFYVFGTGTKVIVLGQPKANPT 120
          *:*********:*:::*:************ ** . :. * :** ***: ******* *:

P001      VTLFPPSSEELQANKATLVCLISDFYPQVTVAWKADSSPVKAGVETTTPSKQSNNKYAAS 179
P011      VTIFPPSSEELQANKATLVCLISDFYPQVTVAWKADGSPVKAGVETTKPSKQSNNKYAAS 180
          **:*********************************.**********.************

P001      SYLSLTPEQWKSHRSYSCQVTHEGSTVEKTVAPTECS 216
P011      SYLSLTPEQWKSHRSYSCQVTHEGSTVEKTVAPTECS 217
          *************************************
```

Figure A.2. Full length sequence alignment of the λ light chains of our study. The framework regions (FR) are marked with blue, the complementarity determining regions (CDRs) with red.

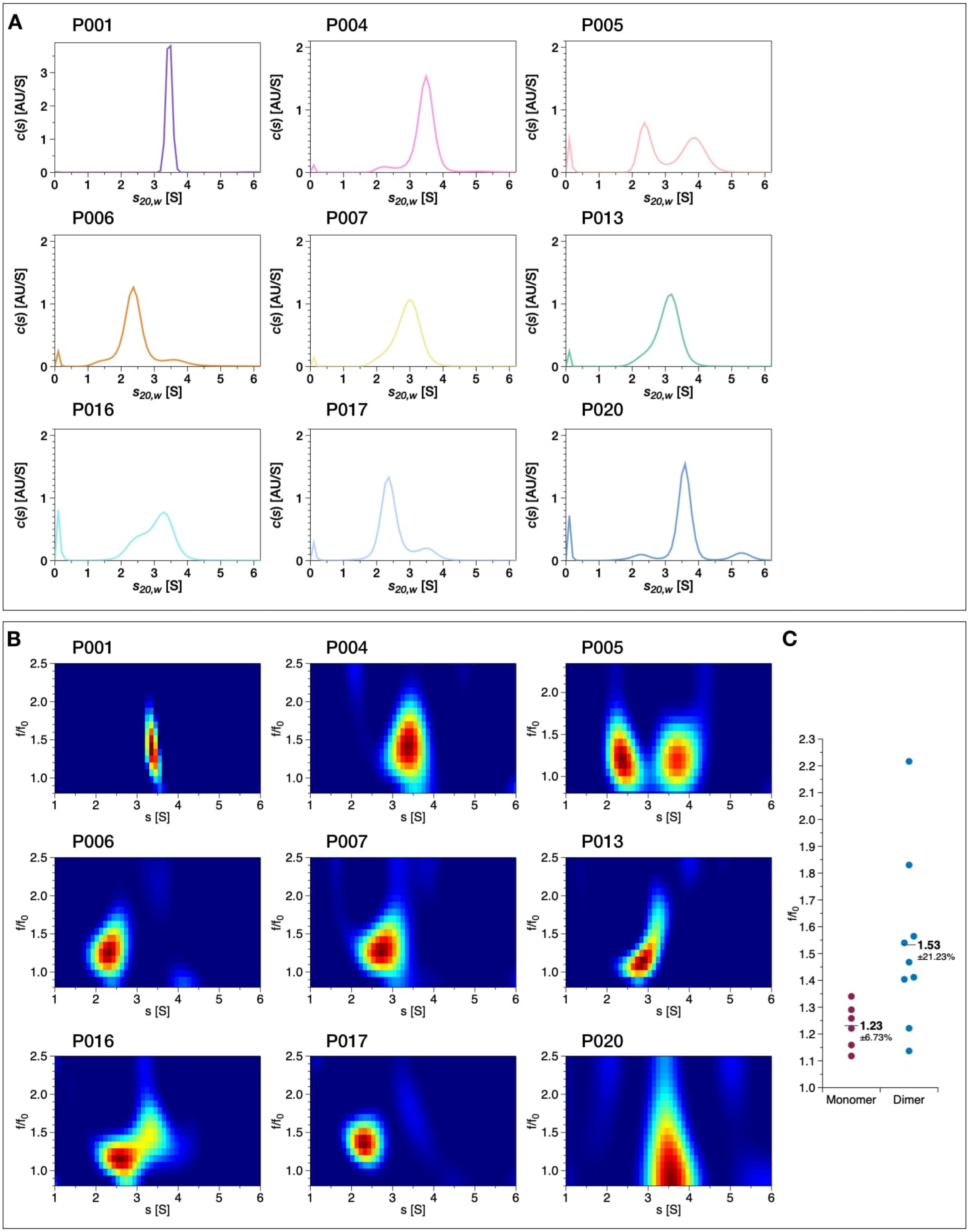

Figure A.3. (A) Distribution of sedimentation coefficients (c(*s*)) of the IgLC samples of this study and (B) application of the c(*s*, f/f_0)-model determined by sedimentation velocity experiments at 60.000 rpm. We find that the form factors of the dimers differ within the sample set. The dimers can appear very globular e.g. P020 with a f/f_0 of 1.14 or elongated such as P006 f/f_0 of 2.22 and P007, P013 and P017 f/f_0 between 1.54 and 1.83. However the signal for the dimer of P006 is very low. (C) shows the variation of f/f_0 between the dimers compared to the monomers. The monomers of P004 and P020 were excluded due to their low signal intensity.

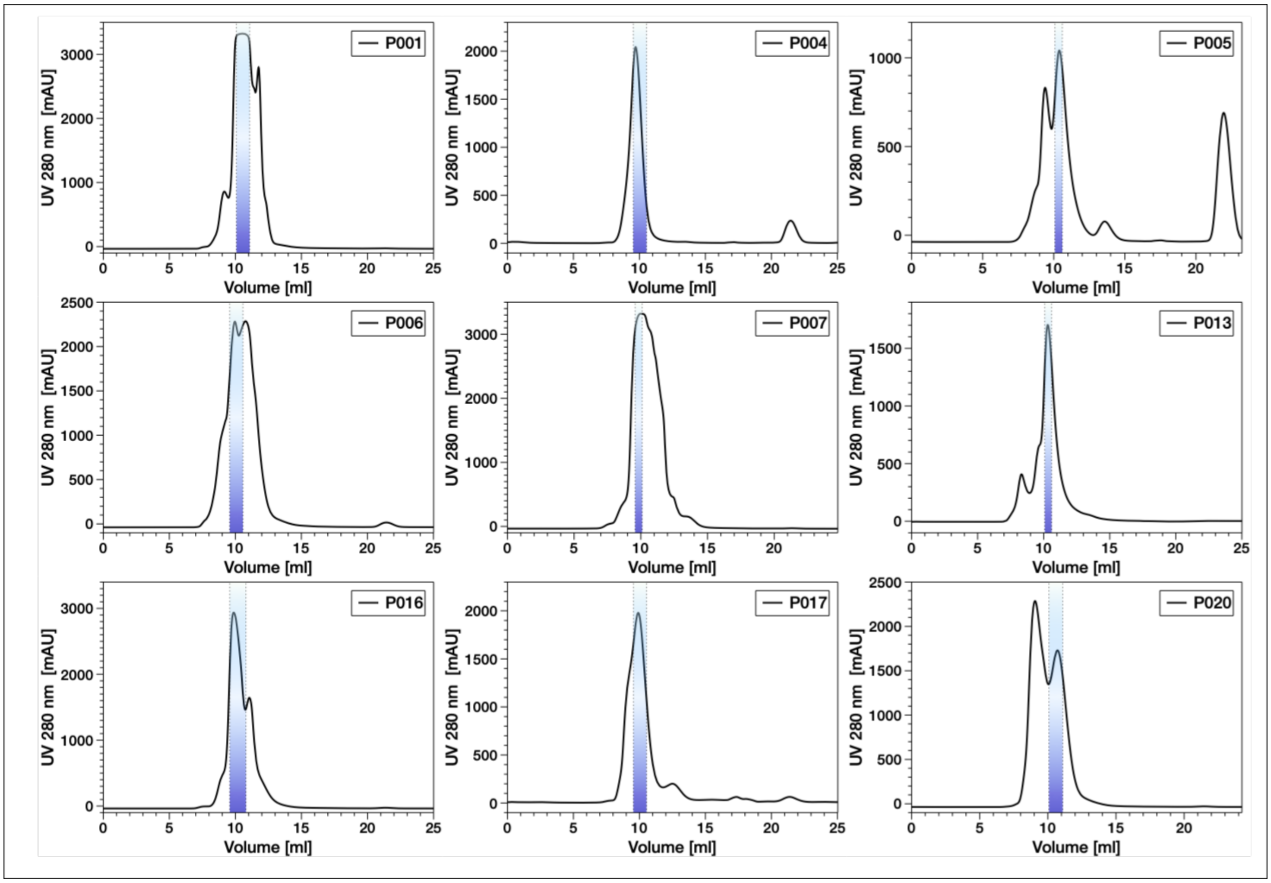

Figure A.4. Size exclusion chromatograms of the different IgLC samples of this study. The fractions used for the remaining experiments in this study are marked in blue
