# Supplementary material for: Widespread amyloidogenicity potential of multiple myeloma patient-derived immunoglobulin light chains
Source: BMC Biol. 2023 Feb 3;21:21. doi: 10.1186/s12915-022-01506-w (PMC9898917; doi:10.1186/s12915-022-01506-w)
Supplement: Supplementary file 2 — Additional file 2: Figure A.1. Concentration dependent thermal unfolding experiments of the IgLC samples of our study. The samples were scanned from 25-70◦C at 1◦C per minute. In each case, the evolution of the ratio of the intrinsic fluorescence intensities at 350 and 330 nm is shown on top, and the evolution of the size distribution, measured by dynamic light scattering (DLS) is shown on a logarithmic scale on the bottom as a contour plot. Contours are spaced with 0.1 and the lowest contour drawn is at 0.03 amplitude. Each sample was measured at 4 concentrations, as the undiluted stock solution as well as 3 dilutions by a factor of 2 each. The concentrations of the stock solutions are 97 μM (P001), 150 μM (P004), 91 μM (P005), 139 μM (P006), 103 μM (P007), 97 μM (P013), 99 μM (P016), 158 μM (P017) and 81 μM (P020). Figure A.2. Temperature-dependent chemical denaturation of the IgLC samples of this study by urea. In each case, the evolution of the ratio of the intrinsic fluorescence intensities at 350 and 330 nm is shown on top, and the evolution of the size distribution, measured by DLS is shown on a logarithmic scale on the bottom as a contour plot. Contours are spaced with 0.1 and the lowest contour drawn is at 0.03 amplitude. The protein concentration corresponds to a 5-fold dilution of the stock solution used for the thermal denaturation in Figure A.1. The urea concentrations are in each case 0, 0.67,1.34, 2.01, 2.68, 3.35, 4.02, 4.69, 5.36 M. The samples were scanned from 20-70◦C at 1◦C per minute. Figure A.3. Global fits of the temperature-dependent chemical denaturation of the IgLC samples of this study by urea. The data is the same as in the previous figure, but instead of using the fluorescence intensity ratio, the fits are performed simultaneously on the fluorescence intensities at both 330 nm (shown here) and 350 nm. Figure A.4. Results from the global fits of the temperature-dependent chemical denaturation of the IgLC samples of this study by urea. Sho [file 12915_2022_1506_MOESM2_ESM.pdf]

A Additional file 2

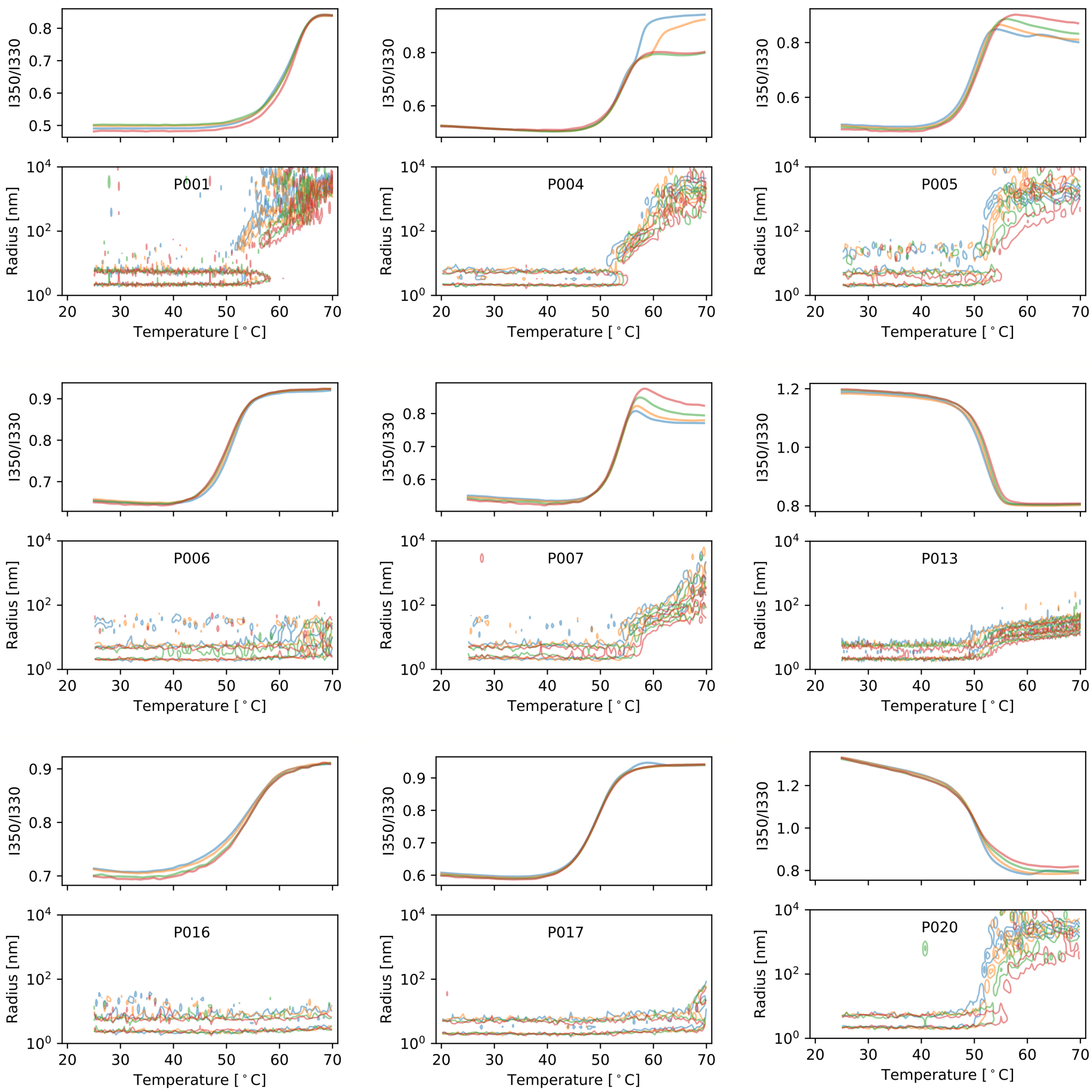

Figure A.1. Concentration dependent thermal unfolding experiments of the IgLC samples of our study. The samples were scanned from 25-70°C at 1°C per minute. In each case, the evolution of the ratio of the intrinsic fluorescence intensities at 350 and 330 nm is shown on top, and the evolution of the size distribution, measured by dynamic light scattering (DLS) is shown on a logarithmic scale on the bottom as a contour plot. Contours are spaced with 0.1 and the lowest contour drawn is at 0.03 amplitude. Each sample was measured at 4 concentrations, as the undiluted stock solution as well as 3 dilutions by a factor of 2 each. The concentrations of the stock solutions are 97 μM (P001), 150 μM (P004), 91 μM (P005), 139 μM (P006), 103 μM (P007), 97 μM (P013), 99 μM (P016), 158 μM (P017) and 81 μM (P020).

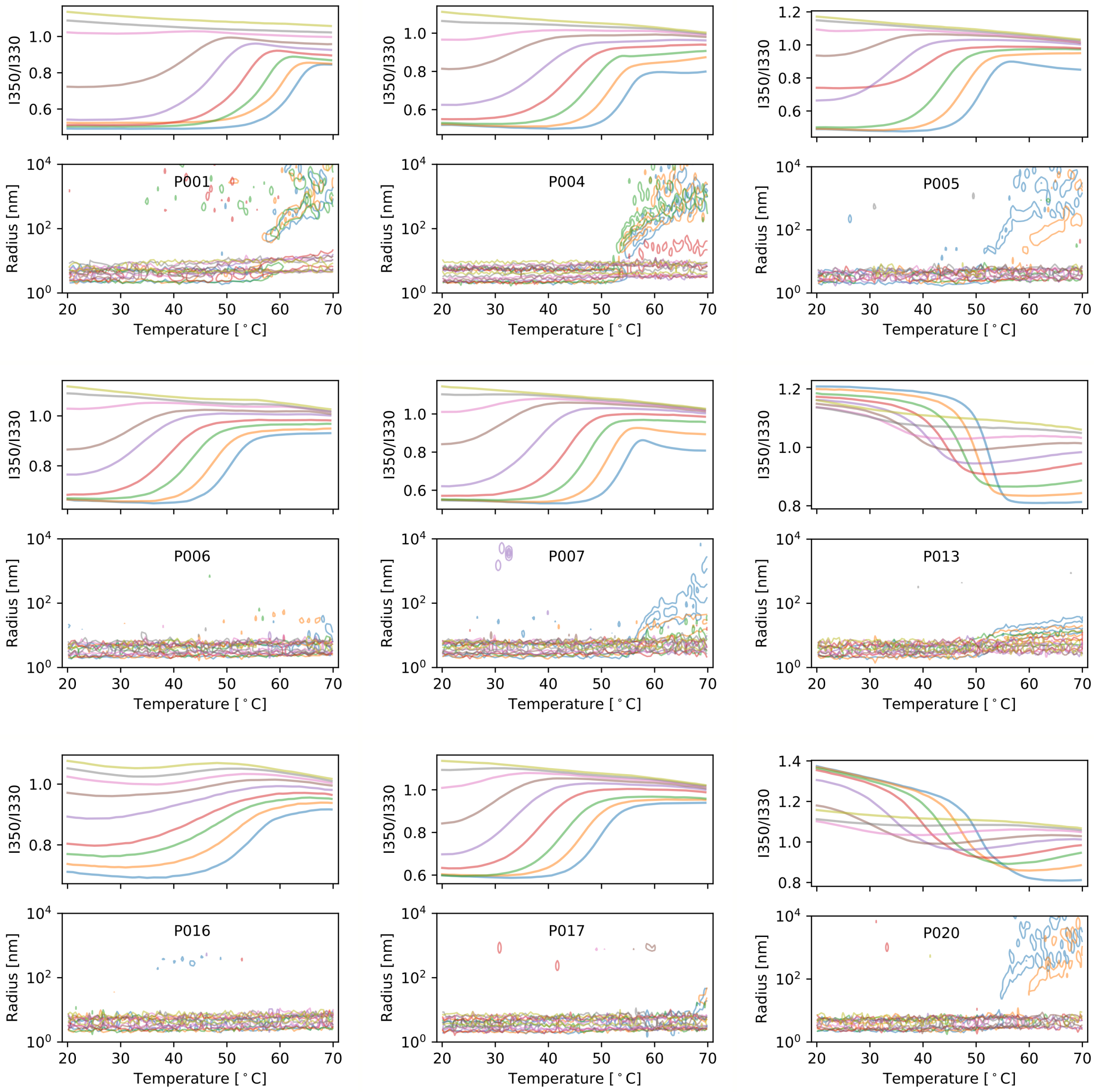

Figure A.2. Temperature-dependent chemical denaturation of the IgLC samples of this study by urea. In each case, the evolution of the ratio of the intrinsic fluorescence intensities at 350 and 330 nm is shown on top, and the evolution of the size distribution, measured by DLS is shown on a logarithmic scale on the bottom as a contour plot. Contours are spaced with 0.1 and the lowest contour drawn is at 0.03 amplitude. The protein concentration corresponds to a 5-fold dilution of the stock solution used for the thermal denaturation in Figure A.1. The urea concentrations are in each case 0, 0.67,1.34, 2.01, 2.68, 3.35, 4.02, 4.69, 5.36 M. The samples were scanned from 20-70°C at 1°C per minute.

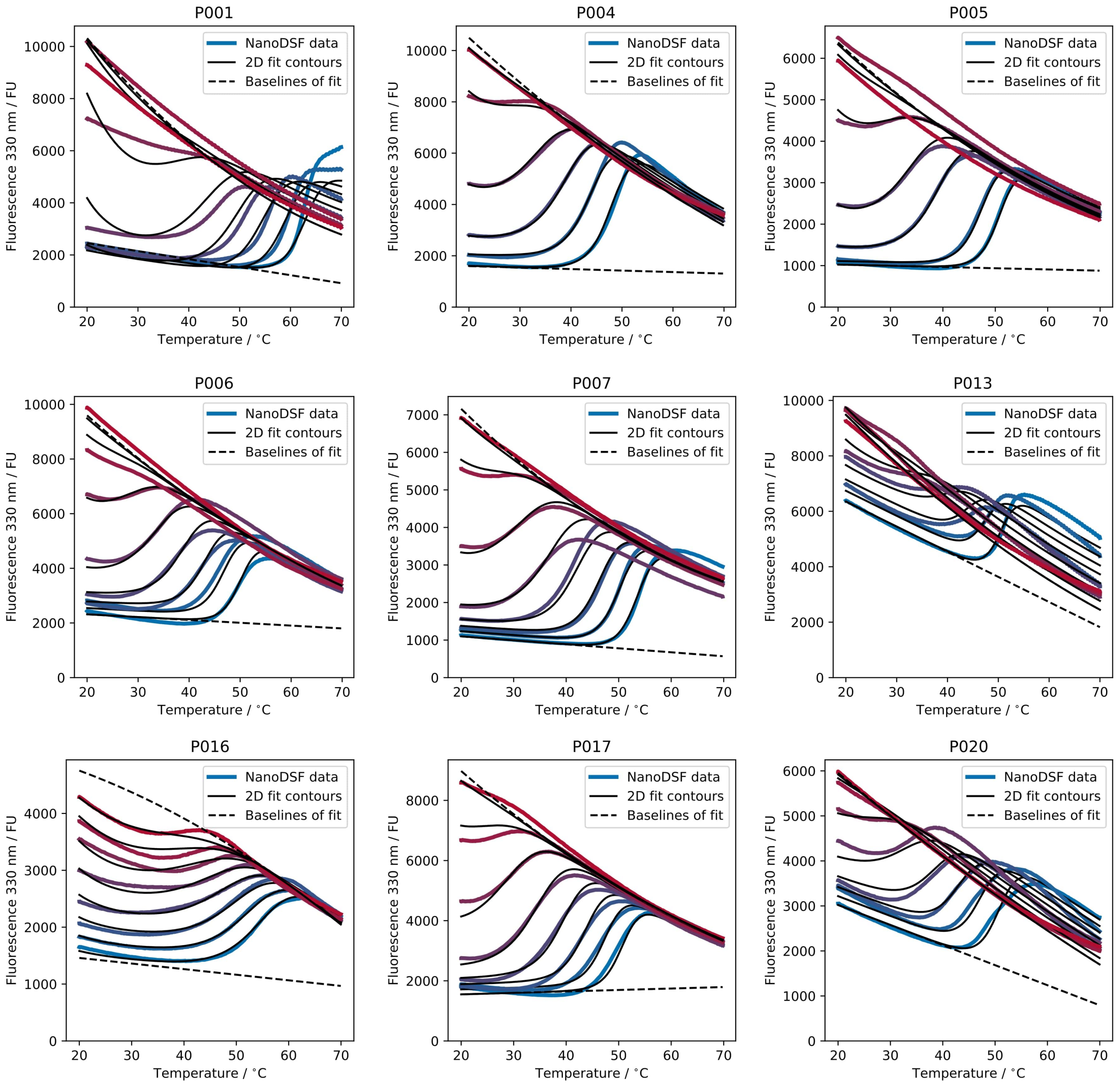

Figure A.3. Global fits of the temperature-dependent chemical denaturation of the IgLC samples of this study by urea. The data is the same as in the previous figure, but instead of using the fluorescence intensity ratio, the fits are performed simultaneously on the fluorescence intensities at both 330 nm (shown here) and 350 nm.

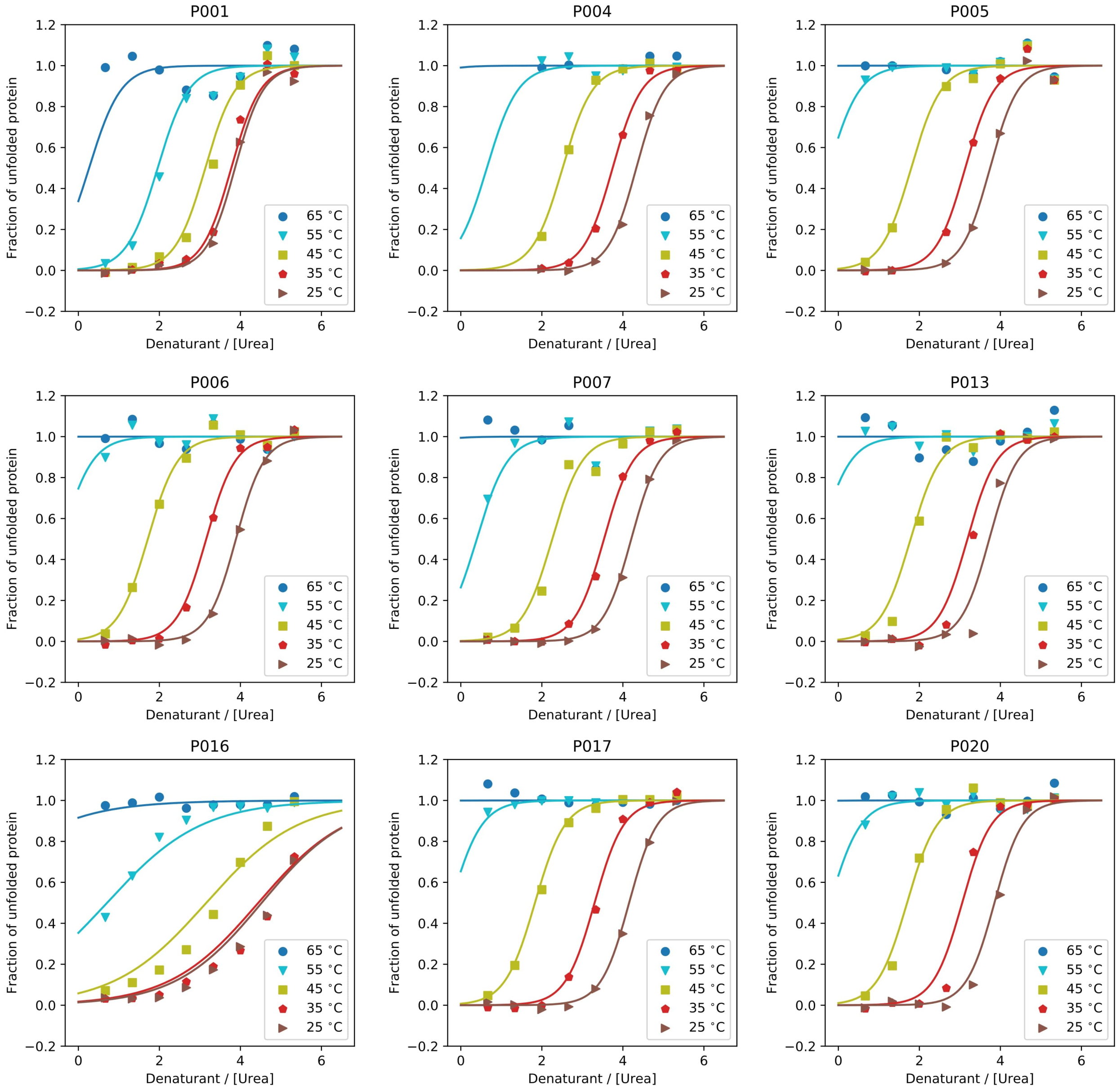

Figure A.4. Results from the global fits of the temperature-dependent chemical denaturation of the IgLC samples of this study by urea. Shown is the fraction of unfolded protein as a function of urea concentration at different temperatures.

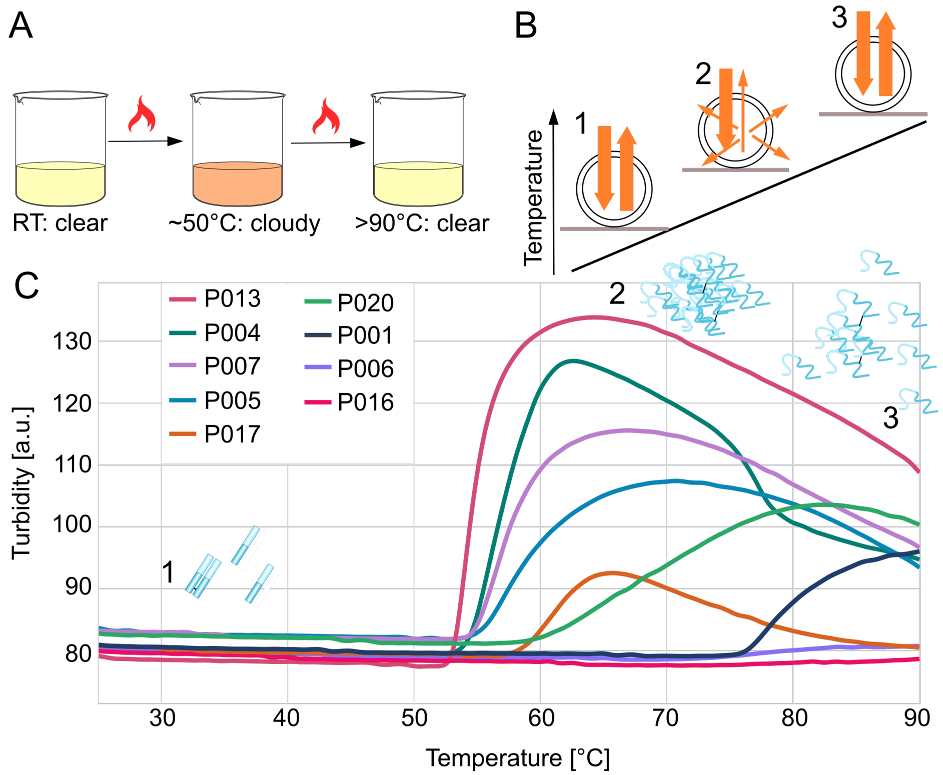

Figure A.5. A modern implementation of the historical test for Bence-Jones proteins. (A) Illustration of the original test for Bence-Jones proteins, i.e. free IgLCs, in urine[?]. After adjustment to pH 5, the urine sample is heated and turns cloudy at approximately 50°C. If further heating to temperatures close to the boiling point leads to a decrease in turbidity, the presence of Bence-Jones proteins is confirmed. (B) Illustration of the BJT in microcapillaries, here shown in cross-section. The sample turbidity is measured with a back-reflection optics. Initially, the IgLCs are in their soluble, monomeric or dimeric form (1). Protein aggregation at intermediate temperatures leads to more scattering and less back-reflection (2), and subsequent heating leads to an increase in back-reflection (3) in the case of typical Bence-Jones behaviour. (C) Sample turbidity is measured as IgLC samples at pH 5 are heated from 25 to 90°C at 2.5°C/min.
