# Supplementary material for: Widespread amyloidogenicity potential of multiple myeloma patient-derived immunoglobulin light chains
Source: BMC Biol. 2023 Feb 3;21:21. doi: 10.1186/s12915-022-01506-w (PMC9898917; doi:10.1186/s12915-022-01506-w)
Supplement: Supplementary file 3 — Additional file 3: Figure A.1. AFM-height-images of aggregates prepared in different reaction vessels. We used as seeds both fibrils which had been prepared in a high-binding surface plate in the presence of glass beads (A), as well as fibrils which had been prepared in the same volume in an 2 ml Eppendorf tube with glass beads (B). The presence of fibrils was confirmed using atomic force microscopy. Although the presence of fibrils could be confirmed in both setups, the total ThT-fluorescence intensity was lower if seeds prepared in an Eppendorf tube were used. The image scale is 5 x 5 μm. The colour range represents the height from -2 to 15 nm. Figure A.2. Aggregation assays of P016 (35 μM monomer concentration) at (A) pH 3 and (B) pH 4 monitored in a high-binding surface plate in the presence of glass beads and conditions of mechanical agitation and with addition of glass beads after 24 h pre-incubation without shaking and the (C) aggregation halftimes (top). Aggregation assays of P016 at (D) pH 3 and (E) pH 4 in a high-binding plate under quiescent conditions. Seeds prepared in a high-binding plate and prepared in an Eppendorf tube are added at the beginning and after 6 h pre-incubation and (F) the halftimes are analysed. The pre-incubation times (24 or 6 h) are subtracted from the halftimes. Figure A.3. The influence of acidic pH on the IgLC samples. (A) Illustration of the finding that acidic pH enables the naturally present proteases in the IgLC samples to cleave the IgLCs into fragments, that are then subsequently forming amyloid fibrils. (B) The fraction of native protein (monomer and dimer combined) at different incubation times determined by SDS-PAGE (left: pH 2, middle: pH 3, right: pH 4) and (C) the hydrodynamic radius in nm (left: pH 3, middle: pH 4) and the normalized concentration measured by Fluidity One (right: pH 4). Figure A.4. (A) Aggregation experiment at 55◦C monitored in a non-binding surface plate under agitation conditions in the presence o [file 12915_2022_1506_MOESM3_ESM.pdf]

A Additional file 3

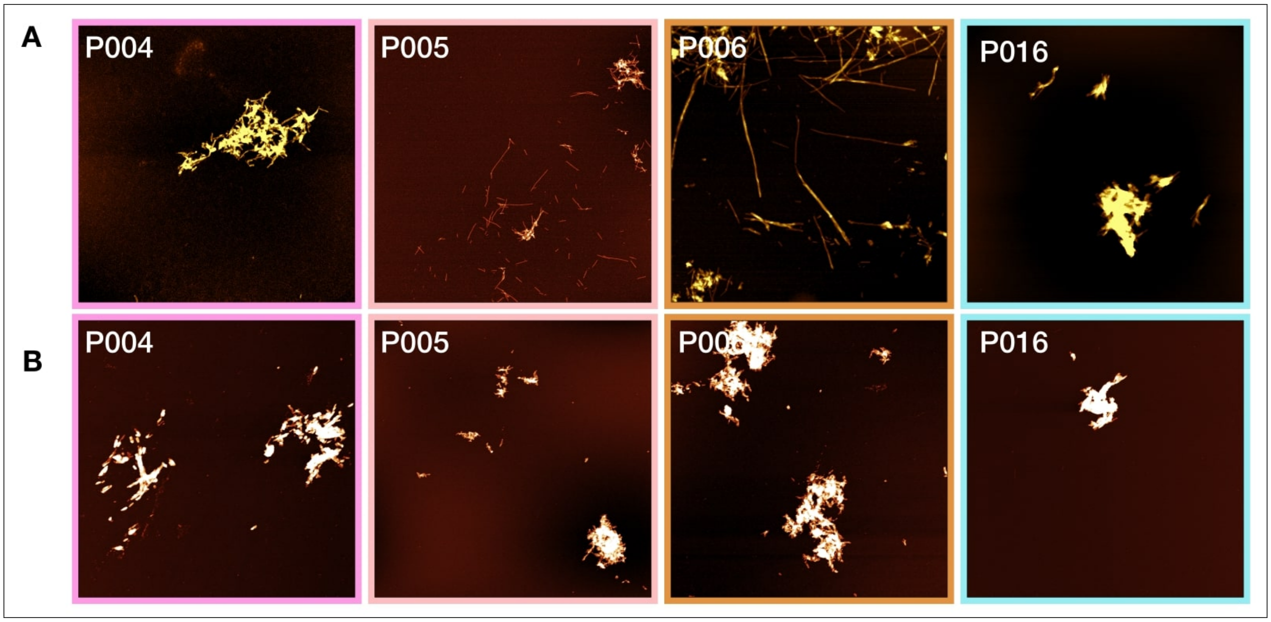

Figure A.1. AFM-height-images of aggregates prepared in different reaction vessels. We used as seeds both fibrils which had been prepared in a high-binding surface plate in the presence of glass beads (A), as well as fibrils which had been prepared in the same volume in an 2 ml Eppendorf tube with glass beads (B). The presence of fibrils was confirmed using atomic force microscopy. Although the presence of fibrils could be confirmed in both setups, the total ThT-fluorescence intensity was lower if seeds prepared in an Eppendorf tube were used. The image scale is 5 x 5 µm. The colour range represents the height from -2 to 15 nm.

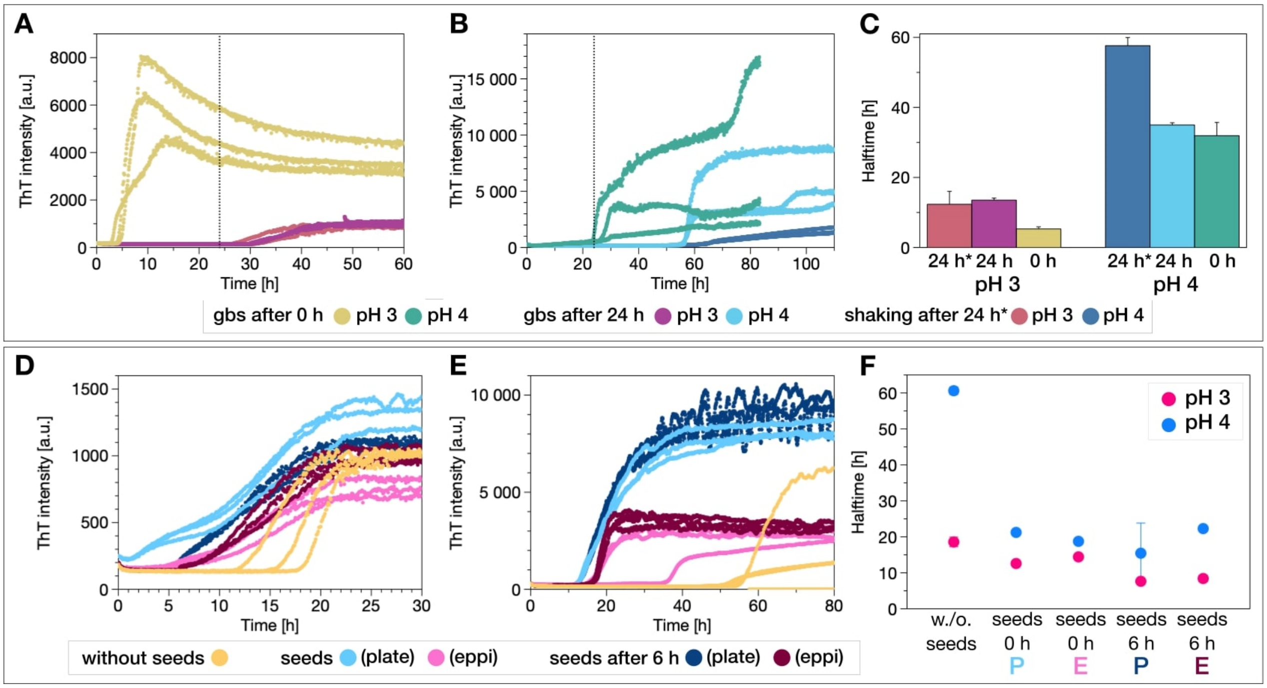

Figure A.2. Aggregation assays of P016 (35 μM monomer concentration) at (A) pH 3 and (B) pH 4 monitored in a high-binding surface plate in the presence of glass beads and conditions of mechanical agitation and with addition of glass beads after 24 h pre-incubation without shaking and the (C) aggregation halftimes (top). Aggregation assays of P016 at (D) pH 3 and (E) pH 4 in a high-binding plate under quiescent conditions. Seeds prepared in a high-binding plate and prepared in an Eppendorf tube are added at the beginning and after 6 h pre-incubation and (F) the halftimes are analysed. The pre-incubation times (24 or 6 h) are subtracted from the halftimes.

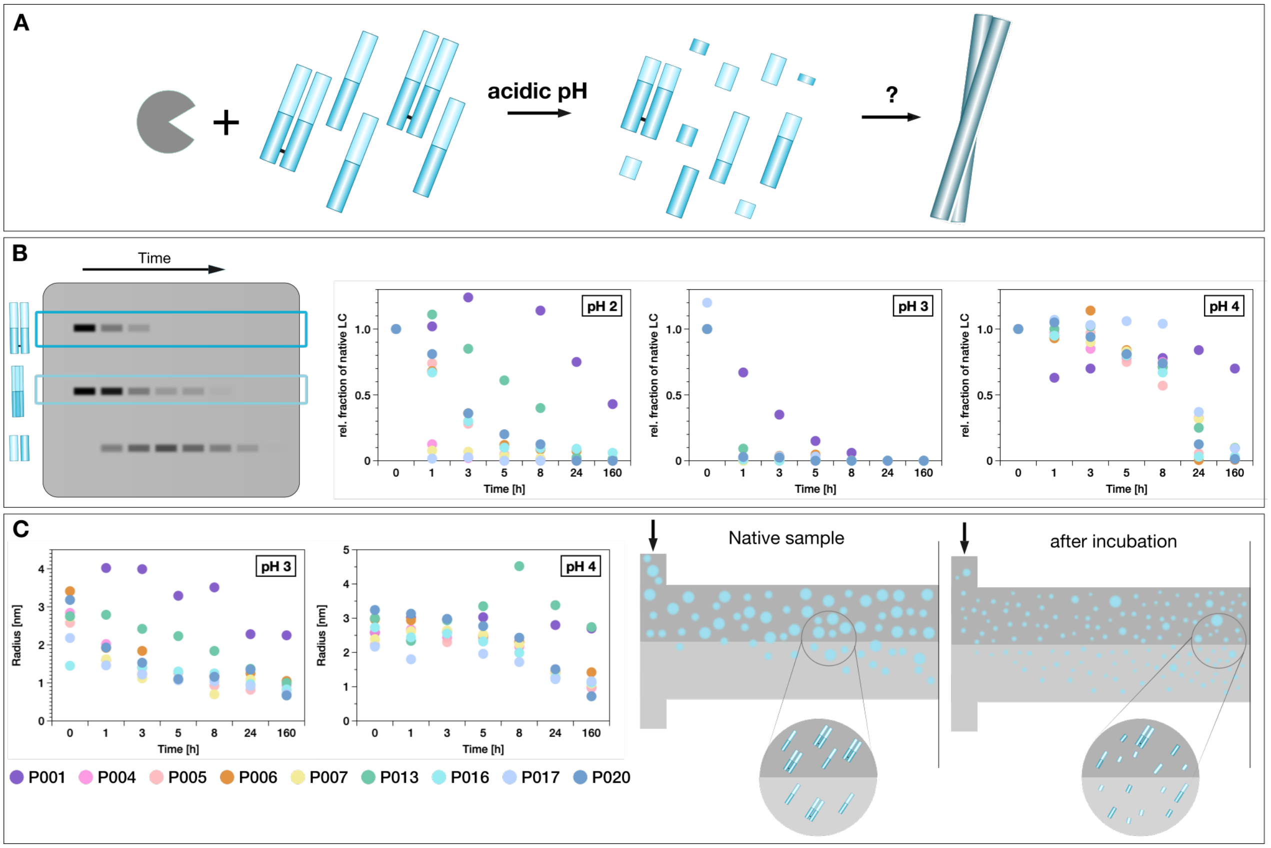

Figure A.3. The influence of acidic pH on the IgLC samples. (A) Illustration of the finding that acidic pH enables the naturally present proteases in the IgLC samples to cleave the IgLCs into fragments, that are then subsequently forming amyloid fibrils. (B) The fraction of native protein (monomer and dimer combined) at different incubation times determined by SDS-PAGE (left: pH 2, middle: pH 3, right: pH 4) and (C) the hydrodynamic radius in nm (left: pH 3, middle: pH 4) and the normalized concentration measured by Fluidity One (right: pH 4).

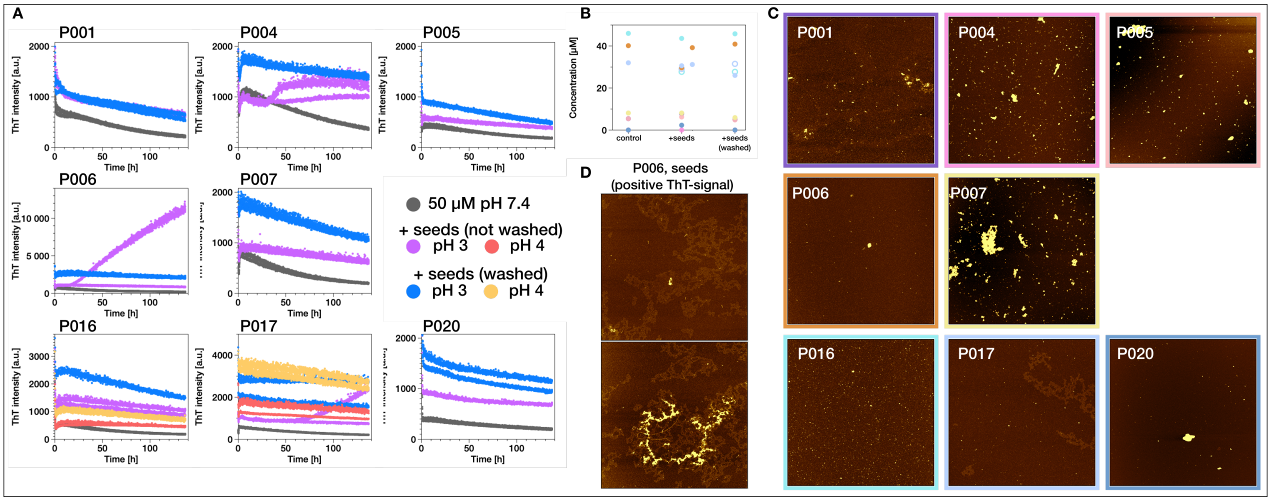

Figure A.4. (A) Aggregation experiment at 55°C monitored in a non-binding surface plate under agitation conditions in the presence of glass beads. 5% Seeds which were produced at pH 3 or pH 4 in an Eppendorf tube were added to 50 μM light chain. (B) The soluble content was determined after the experiment by UV-absorbance. AFM-height-images of the light chains at the end of the experiments (C) and of aggregated P006 with the positive ThT-signal (D). Fibrils at pH 3 do not display any twist. The image scale is 5 x 5 μm. The colour range represents the height from -2 to 5 nm.

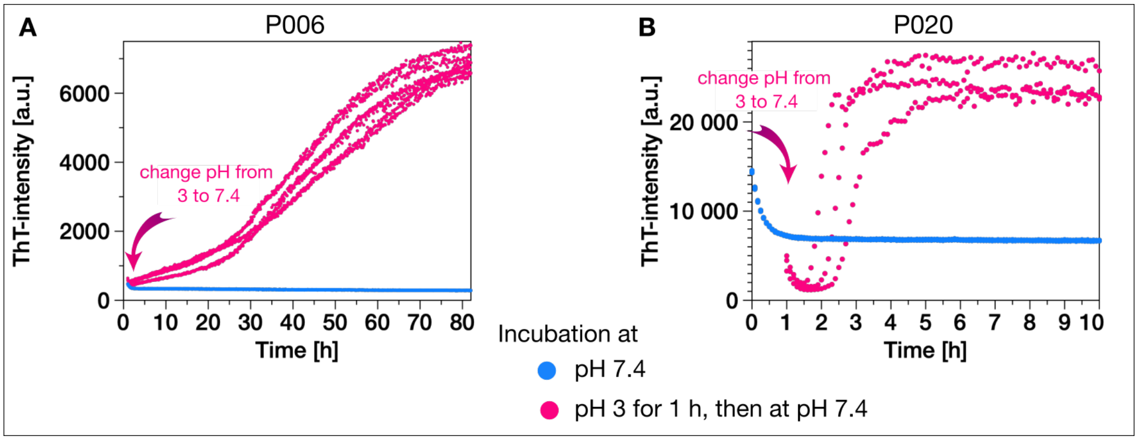

Figure A.5. Aggregation experiment of (A) P006 and (B) P020. After pre-incubation at pH 3 under quiescent conditions at 37°C, the sample was diluted with 1 M Tris-HCl, pH 11.1, to achieve a pH of 7.4 and a protein concentration of 35 μM (pink). The aggregation was monitored in a high-binding surface plate under agitation conditions in the presence of glass beads. The negative control without pre-incubation (blue) showed no increase in ThT-fluorescence over a time course of 120 h.

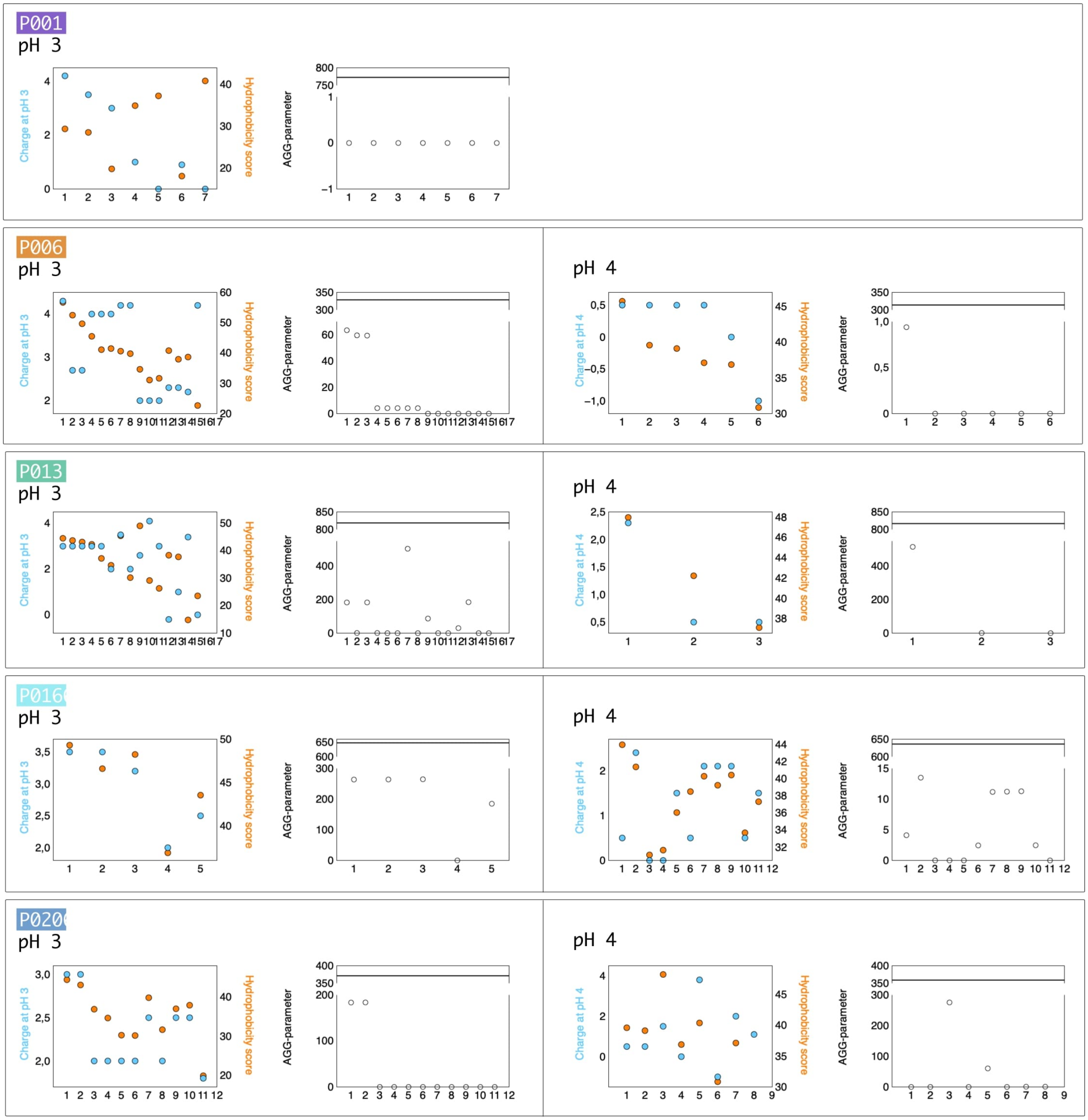

Figure A.6. The sequence regions of P001, P006, P013, P016 and P020 that were found in the aggregates formed at acidic pH values and identified by mass spectrometry. The sequence regions were analysed according to their charge at the depicted pH value (blue) and hydrophobicity score (orange) using the Peptide Analyzing Tool from Thermofisher. The hydrophobicity score is based on the index proposed by Krokhin and Spicer[?]. The AGG parameters of the sequence fragments were analysed using the TANGO algorithm, the horizontal line indicates the AGG parameter value of the full length sequence.
