# Supplementary material for: Widespread amyloidogenicity potential of multiple myeloma patient-derived immunoglobulin light chains
Source: BMC Biol. 2023 Feb 3;21:21. doi: 10.1186/s12915-022-01506-w (PMC9898917; doi:10.1186/s12915-022-01506-w)
Supplement: Supplementary file 4 — Additional file 4: Figure A.1. The IgLC sequences P004, P005, P007, P011 and P017 were analysed with different amyloid prediction tools and the predicted aggregation hot spots visualised on the sequence. Amyloid propensity was determined with the algorithm PASTA (red pH 3, blue pH 4) and the amyloidogenic sequence regions with the algorithms TANGO and WALTZ. The sequence parts which were found in the aggregates formed at acidic pH values are marked with black lines. Figure A.2. (A) Intrinsic solubility score measured by CamSol and (B) aggregation parameter determined by the Tango algorithm at pH 3, pH 4 and pH 7, (C) aggregation free energy computed with the Pasta algorithm. Figure A.3. The κ IgLC sequences P004, P005, P006 and P007 were analysed with different amyloid prediction tools and the results visualised on the sequence. Rosetta energy was determined by ZipperDB, amyloidogenic sequence regions with the algorithms Waltz, Tango and Pasta. The analyses with Tango were performed at pH 3 (red), pH 4 (green) and pH 7 (blue). Figure A.4. The κ IgLC sequences P013, P016, P017 and P020 were analysed with different amyloid prediction tools and visualised on the sequence. Rosetta energy was determined by ZipperDB, amyloidogenic sequence regions with the algorithms Waltz, Tango and Pasta. The analyses with Tango were performed at pH 3 (red), pH 4 (green) and pH 7 (blue). Figure A.5. The λ IgLC sequences P001 and P011 were analysed with different amyloid prediction tools and visualised on the sequence. Rosetta energy was determined by ZipperDB, amyloidogenic sequence regions with the algorithms Waltz, Tango and Pasta. The analyses with Tango were performed at pH 3 (red), pH 4 (green) and pH 7 (blue). Figure A.6. Variable κ region sequences were analysed with Tango at pH 7 (A) and pH 3 (B). The sequences, which originate from different germline sequences were selected from AL-Base and were categorised according to whether the patient was suffering from AL-Amyloidosis or M [file 12915_2022_1506_MOESM4_ESM.pdf]

# A Additional file 4

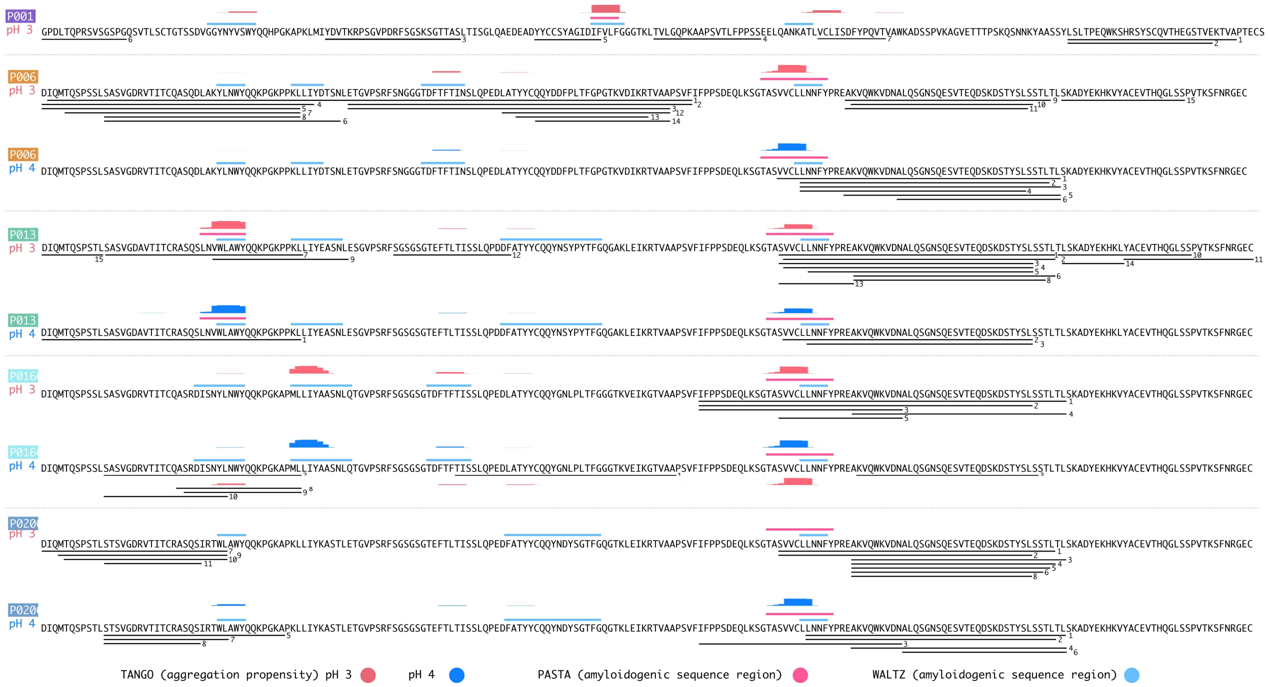


**Figure A.1.** The IgLC sequences P004, P005, P007, P011 and P017 were analysed with different amyloid prediction tools and the predicted aggregation hot spots visualised on the sequence. Amyloid propensity was determined with the algorithm PASTA (red pH 3, blue pH 4) and the amyloidogenic sequence regions with the algorithms TANGO and WALTZ. The sequence parts which were found in the aggregates formed at acidic pH values are marked with black lines.

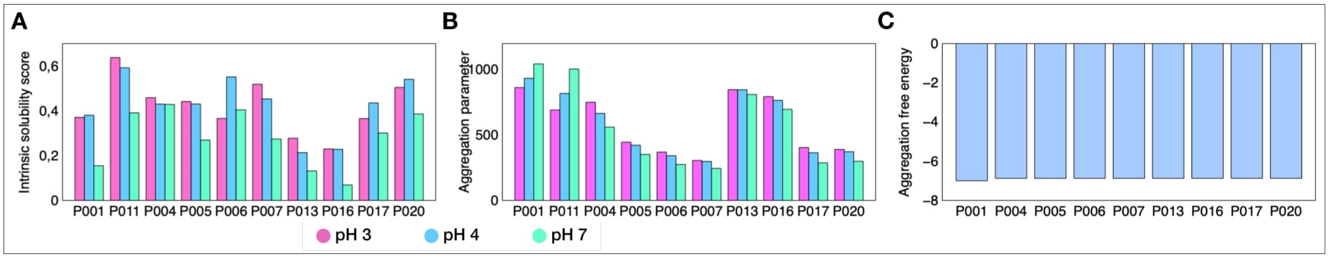


**Figure A.2.** (A) Intrinsic solubility score measured by CamSol and (B) aggregation parameter determined by the Tango algorithm at pH 3, pH 4 and pH 7, (C) aggregation free energy computed with the Pasta algorithm.

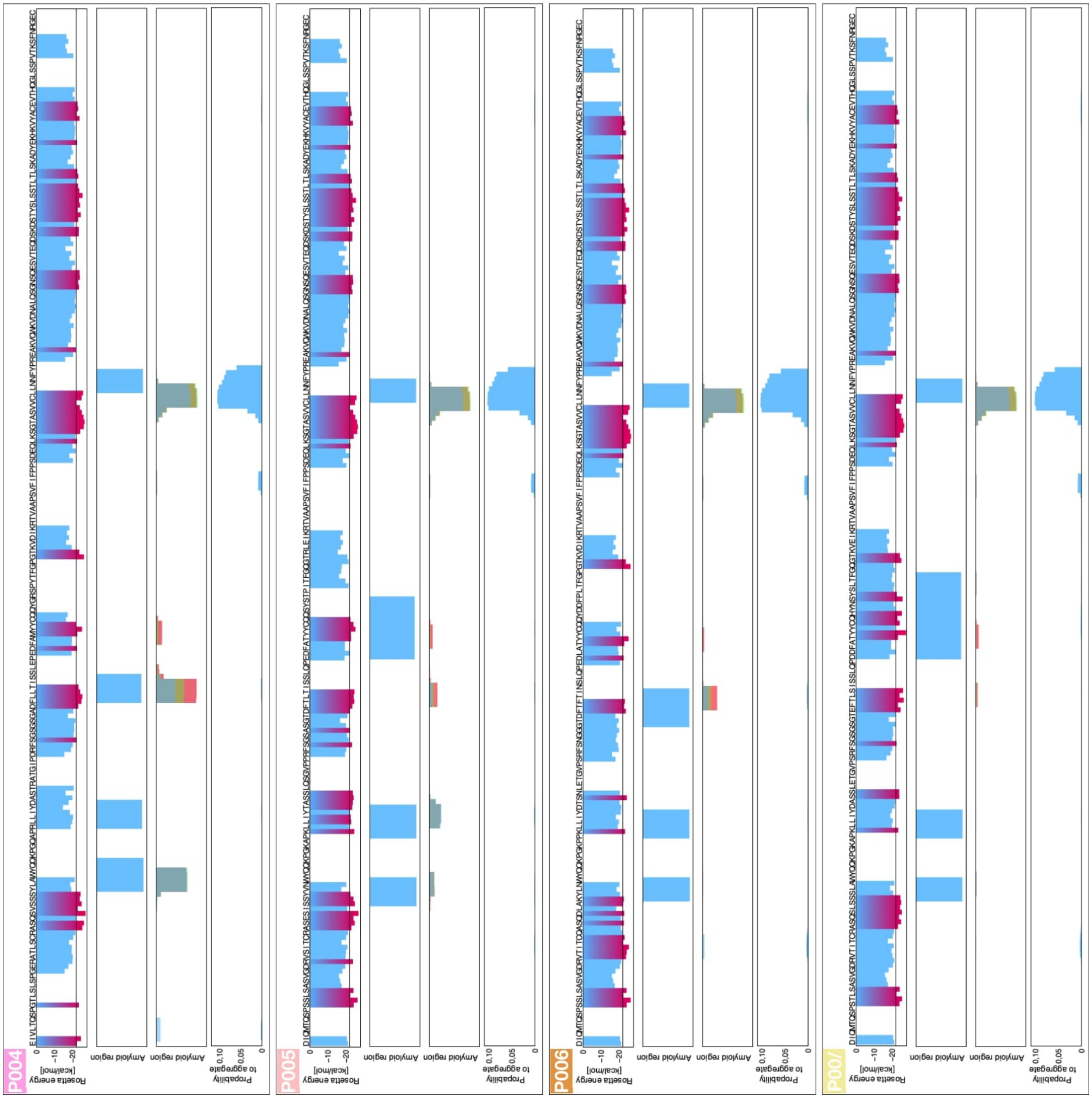


**Figure A.3.** The $\kappa$ IgLC sequences P004, P005, P006 and P007 were analysed with different amyloid prediction tools and the results visualised on the sequence. Rosetta energy was determined by ZipperDB, amyloidogenic sequence regions with the algorithms Waltz, Tango and Pasta. The analyses with Tango were performed at pH 3 (red), pH 4 (green) and pH 7 (blue).

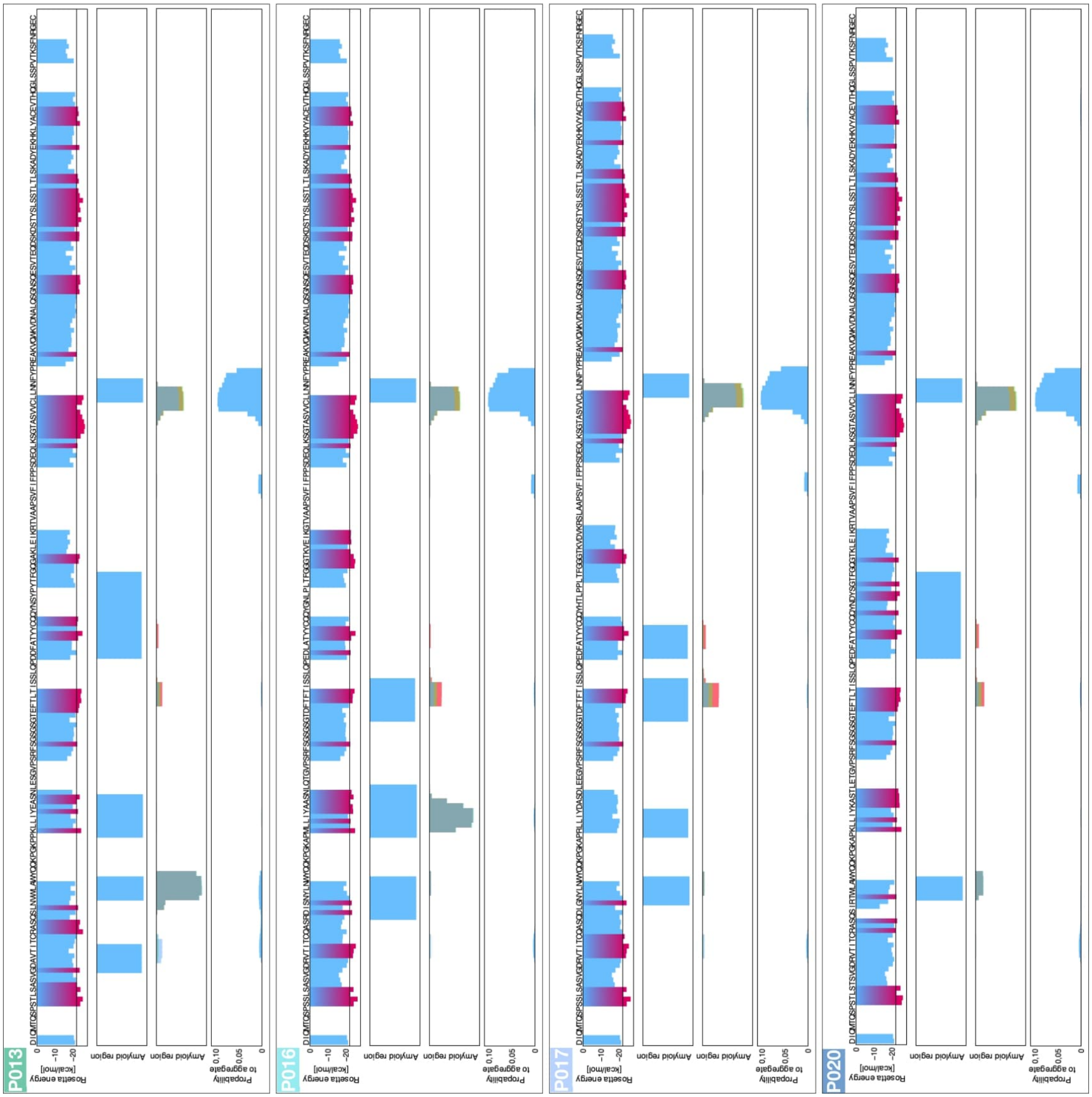


**Figure A.4.** The $\kappa$ IgLC sequences P013, P016, P017 and P020 were analysed with different amyloid prediction tools and visualised on the sequence. Rosetta energy was determined by ZipperDB, amyloidogenic sequence regions with the algorithms Waltz, Tango and Pasta. The analyses with Tango were performed at pH 3 (red), pH 4 (green) and pH 7 (blue).

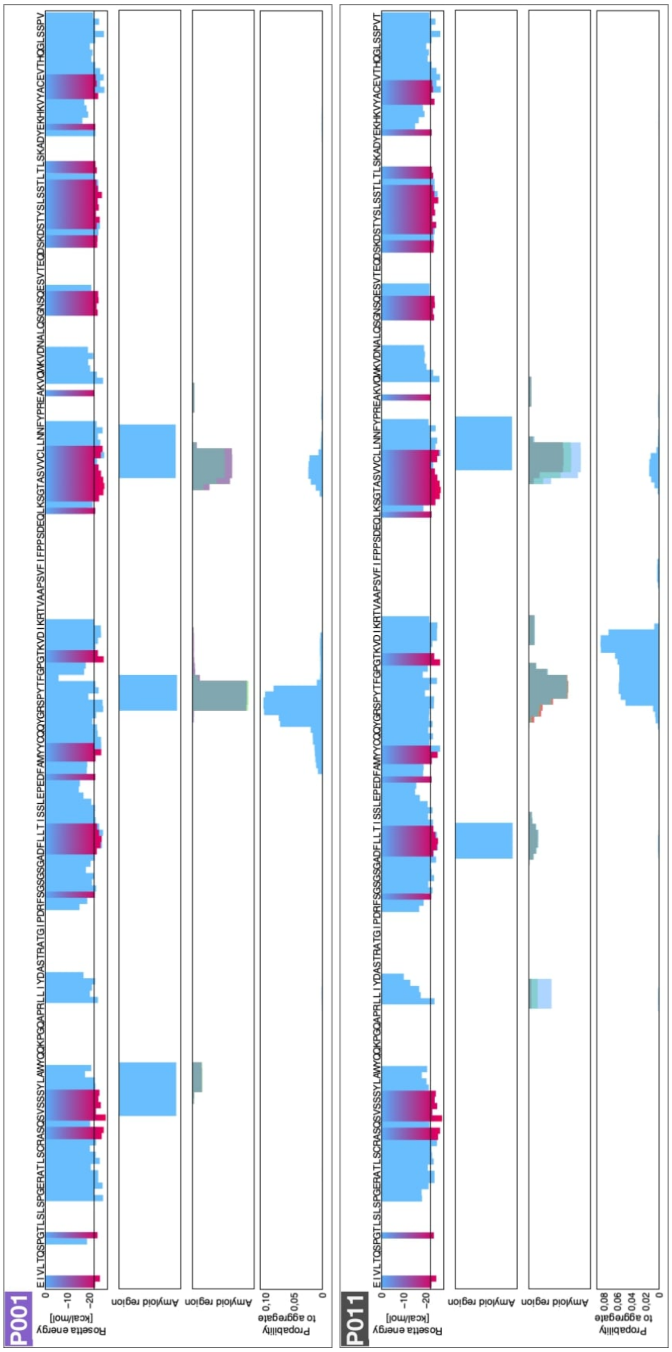


**Figure A.5.** The $\lambda$ IgLC sequences P001 and P011 were analysed with different amyloid prediction tools and visualised on the sequence. Rosetta energy was determined by ZipperDB, amyloidogenic sequence regions with the algorithms Waltz, Tango and Pasta. The analyses with Tango were performed at pH 3 (red), pH 4 (green) and pH 7 (blue).

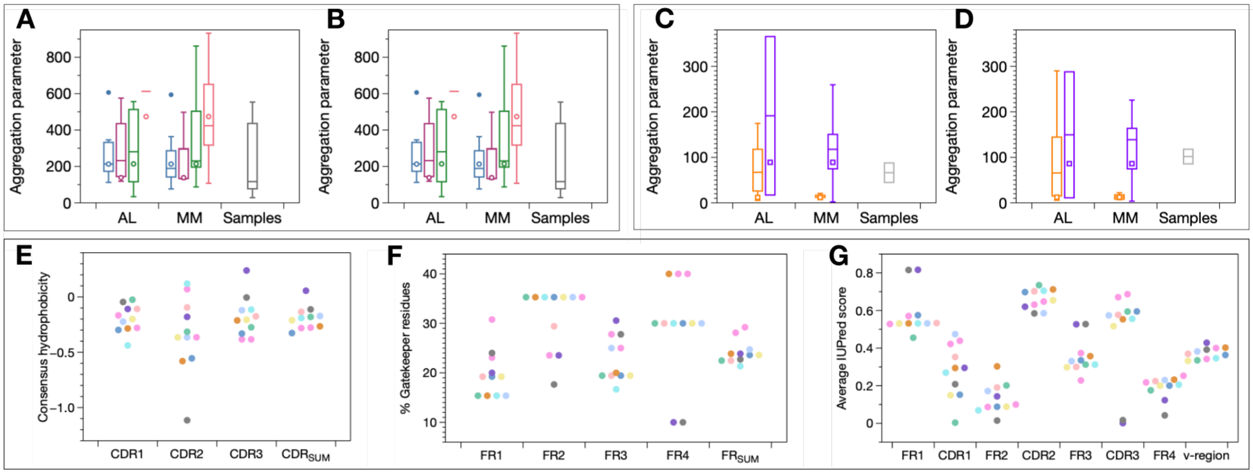


**Figure A.6.** Variable $\kappa$ region sequences were analysed with Tango at pH 7 (A) and pH 3 (B). The sequences, which originate from different germline sequences were selected from AL-Base and were categorised according to whether the patient was suffering from AL-Amyloidosis or MM. IGKV1-5 (blue) AL n=10, MM n=9; IGKV1-33 (violet) AL n=8, MM n=10; IGKV1-39 (green) AL n=10, MM n=10; IGKV3-20 (orange) AL n=7, MM n=10. Variable lambda region sequences were analysed with Tango at pH 7 (C) and pH 3 (D). The sequences, which originate from different germline sequences were selected from AL-Base and were categorised according to whether the patient was suffering from AL-Amyloidosis or MM. IGLV1-40 (orange) AL n=10, MM n=5; IGLV2-11 (violet) AL n=2, MM n=11. The open circles indicate the germline sequence. The aggregation related features according to[?] (E) consensus hydrophopbicity of the CDRs, (F) percentage of the gatekeeper residues in FRs and (G) average IUPred score calculated for the different segments of the variable region of investigated IgLCs. The colour code is equivalent to the previous figures.
