# Supplementary material for: Widespread amyloidogenicity potential of multiple myeloma patient-derived immunoglobulin light chains
Source: BMC Biol. 2023 Feb 3;21:21. doi: 10.1186/s12915-022-01506-w (PMC9898917; doi:10.1186/s12915-022-01506-w)
Supplement: Supplementary file 5 — Additional file 5: Figure A.1. Pearson correlation matrix between all experimental parameters of this study and clinical patient descriptors. The number of data points for each separate correlation can be seen in Figure 3 below. Figure A.2. Spearman correlation matrix between all experimental parameters of this study and clinical patient descriptors. The number of data points for each separate correlation can be seen in Figure 3 below. Figure A.3. Scatter matrix that shows the pairwise correlation plots in the off-diagonal elements, and a histogram of the parameter values in the diagonal elements. Figure A.4. An elastic net model was trained to predict (GFR-CKD-EPI) using all the biophysical parameters measured of the light chains. a) Negative mean squared errors of the models are shown for training and test sets using 4-fold cross validation as a function of the regularisation parameters. No data points were omitted as a separate validation set because of the low amount of data. b) The best model with Alpha = 3.98 and L1Ratio = 1.0 is shown. This model uses only the three parameters trypsin digestibility, ΔG, and dTagg/dlog c, with a Pearson correlation of 0.91. The mean error of cross validation of the model is 18.97±12.14. The influence of dTagg/dlog c seems to mainly arise from a single data point, so the two other parameters are probably more robust. This type of behaviour emphasises the need for significantly larger data sets than the ones that are currently available. Searching for correlations within combinations of parameters provides a large freedom that needs to be constrained by the size of the data set. [file 12915_2022_1506_MOESM5_ESM.pdf]

A Additional file 5

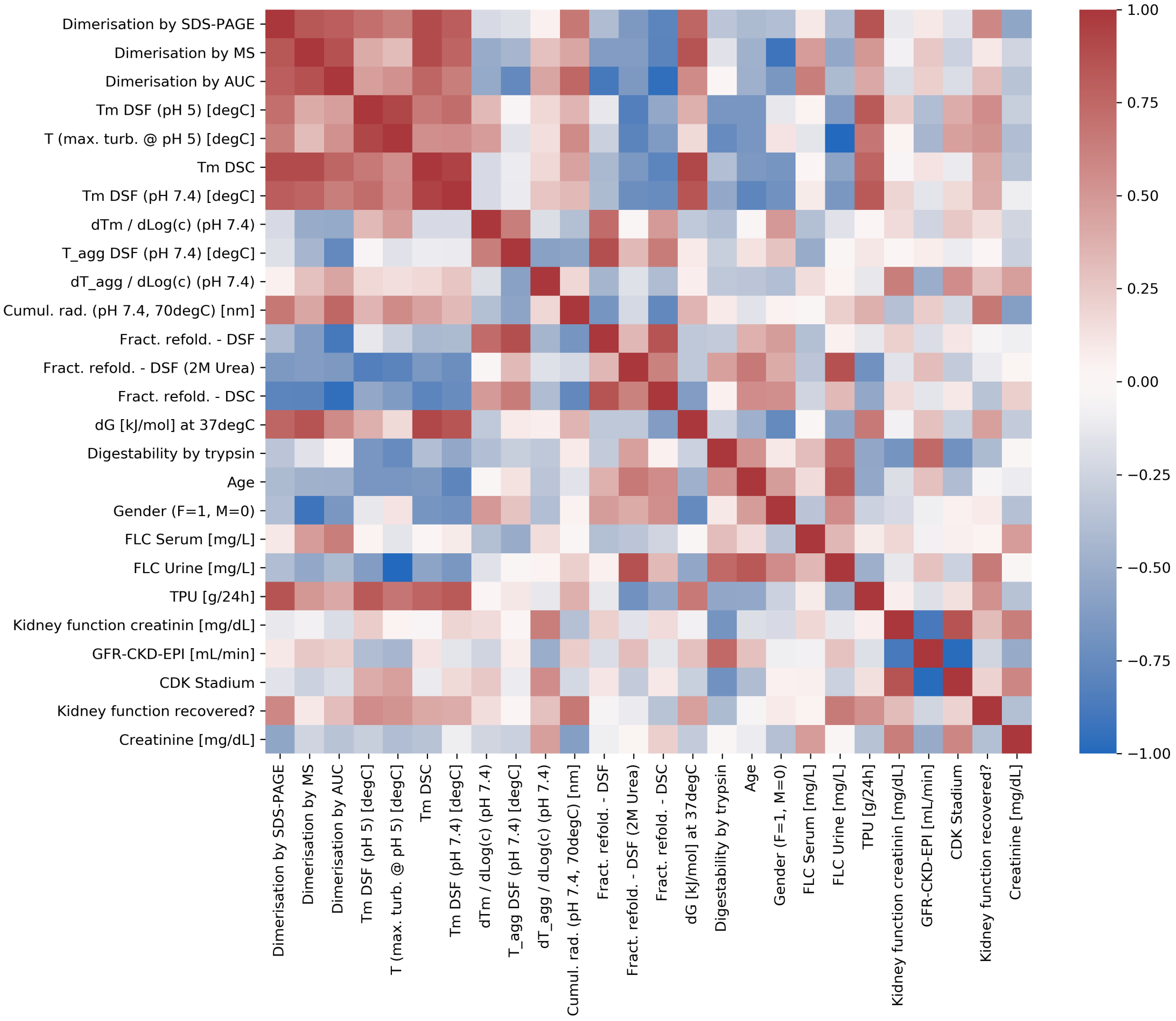

Figure A.1. Pearson correlation matrix between all experimental parameters of this study and clinical patient descriptors. The number of data points for each separate correlation can be seen in Figure 3 below.

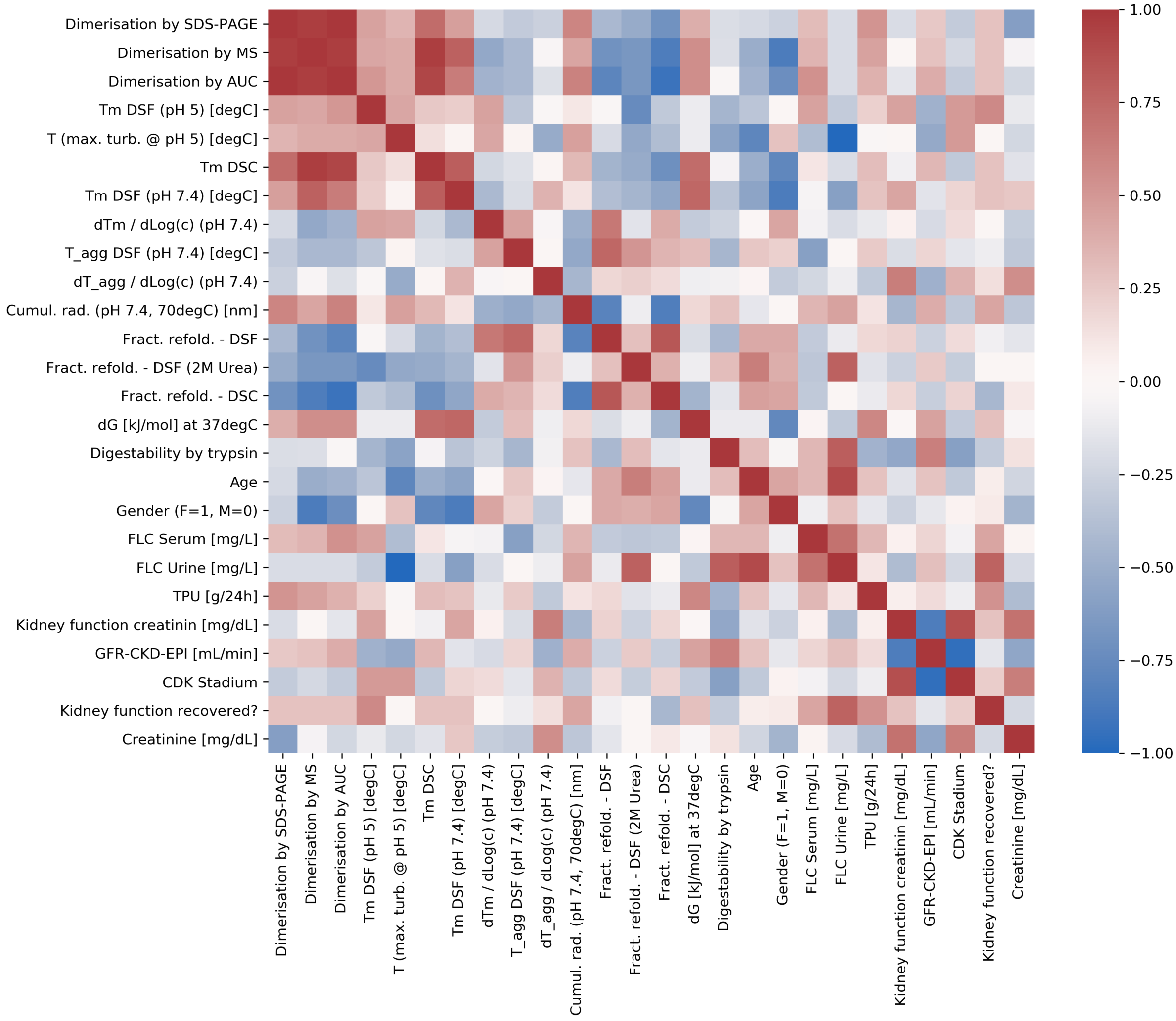

Figure A.2. Spearman correlation matrix between all experimental parameters of this study and clinical patient descriptors. The number of data points for each separate correlation can be seen in Figure 3 below.

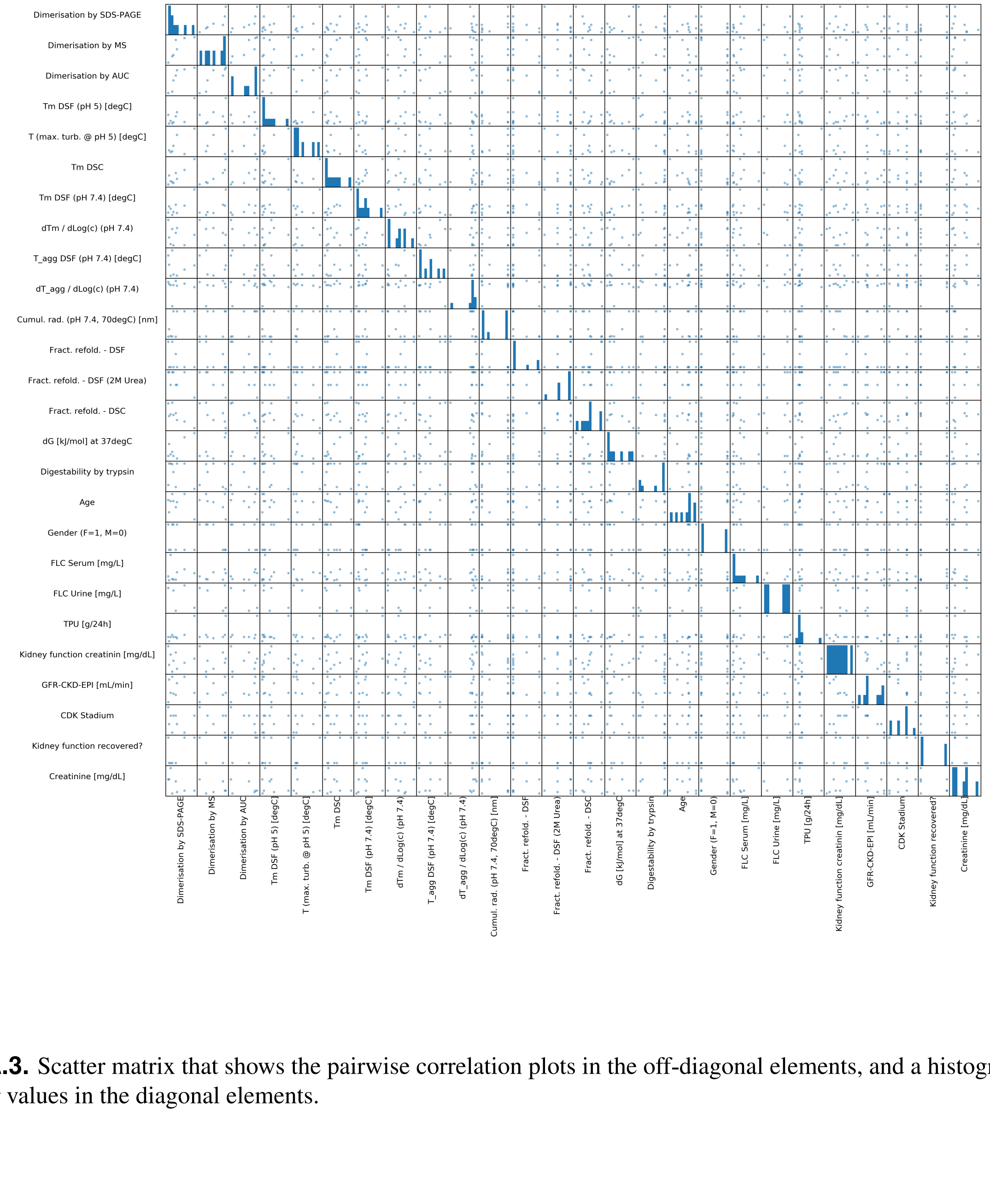

Figure A.3. Scatter matrix that shows the pairwise correlation plots in the off-diagonal elements, and a histogram of the parameter values in the diagonal elements.

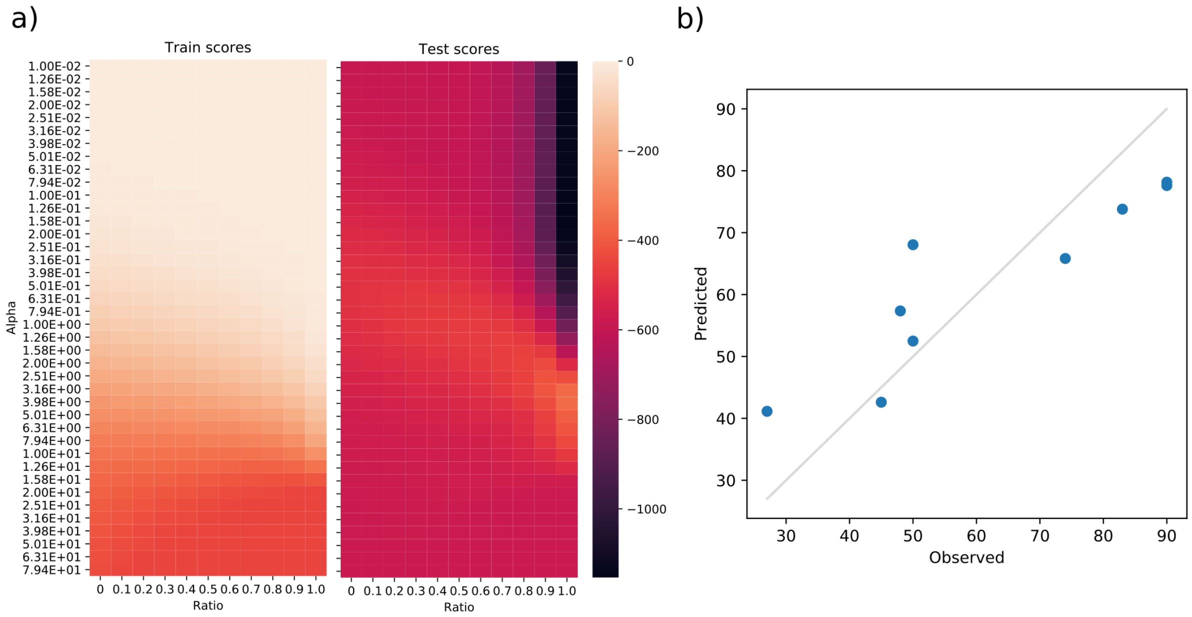

Figure A.4. An elastic net model was trained to predict (GFR-CKD-EPI) using all the biophysical parameters measured of the light chains. a) Negative mean squared errors of the models are shown for training and test sets using 4-fold cross validation as a function of the regularisation parameters. No data points were omitted as a separate validation set because of the low amount of data. b) The best model with $Alpha = 3.98$ and $L1Ratio = 1.0$ is shown. This model uses only the three parameters trypsin digestibility, ΔG, and $\mathrm{d}T_{agg}/\mathrm{dlog}\,c$, with a Pearson correlation of 0.91. The mean error of cross validation of the model is 18.97 ± 12.14. The influence of $\mathrm{d}T_{agg}/\mathrm{dlog}\,c$ seems to mainly arise from a single data point, so the two other parameters are probably more robust. This type of behaviour emphasises the need for significantly larger data sets than the ones that are currently available. Searching for correlations within combinations of parameters provides a large freedom that needs to be constrained by the size of the data set.
